# Quality of Care Transition for COVID-19 Patients in a University Hospital in Southern Brazil

**DOI:** 10.1590/0034-7167-2023-0402

**Published:** 2024-06-28

**Authors:** Ana Cecilia Boeng, Caroline Cechinel-Peiter, Maria Fernanda Baeta Neves Alonso da Costa, Laísa Fisher Wachholz, José Luís Guedes dos Santos, Gabriela Marcellino de Melo Lanzoni

**Affiliations:** I Universidade Federal de Santa Catarina. Florianópolis, Santa Catarina, Brazil

**Keywords:** Transitional Care, COVID-19, Patient Discharge, Continuity of Patient Care, Hospitals, University, Cuidado de Transición, COVID-19, Alta del Paciente, Continuidad de la Atención al Paciente, Hospitales Universitarios

## Abstract

**Objective:**

To assess the quality of care transition from hospital to home for COVID-19 patients.

**Method:**

A cross-sectional study conducted at a University Hospital in Southern Brazil, involving 78 patients discharged after COVID-19 hospitalization. Data collection was performed via telephone using the Brazilian version of the Care Transitions Measure (CTM-15). Data were analyzed using descriptive and analytical statistics.

**Results:**

The mean quality of care transition was 70.8 on a scale ranging from zero to 100, indicating moderate quality of care transition. The highest score was attributed to factor 1, “Preparation for self-management,” and the lowest to factor 4, “Care Plan.”

**Conclusions:**

It is important to enhance communication and support provided to patients during the transition process, especially regarding understanding prescribed medications and the development of clear care plans.

## RESUMEN

**Objetivo:**

Evaluar la calidad de la transición del cuidado desde el hospital hasta el hogar para pacientes con COVID-19.

**Método:**

Estudio transversal realizado en un Hospital Universitario del sur de Brasil, que involucró a 78 pacientes dados de alta después de la hospitalización por COVID-19. La recolección de datos se realizó por teléfono utilizando la versión brasileña de la Medida de Transiciones del Cuidado (CTM-15). Los datos se analizaron utilizando estadísticas descriptivas y analíticas.

**Resultados:**

La calidad media de la transición del cuidado fue de 70.8 en una escala que va de cero a 100, lo que indica una calidad moderada de la transición del cuidado. La puntuación más alta se atribuyó al factor 1, “Preparación para el autogestionamiento”, y la más baja al factor 4, “Plan de cuidado”.

**Conclusiones:**

Es importante mejorar la comunicación y el apoyo proporcionado a los pacientes durante el proceso de transición, especialmente en lo que respecta a la comprensión de los medicamentos recetados y el desarrollo de planes de cuidado claros.

## INTRODUCTION

The pandemic has disrupted the capacity of healthcare systems, resulting in drastic consequences in the social, economic, and public health domains^(1)^. Prior to the pandemic, the transition between healthcare services was already a period susceptible to disruptions in continuity of care^(2)^. The hospital discharge process, likewise, is complex, and the transition of the patient to home represents the moment when the patient is most susceptible to adverse events^(3)^.

The health crisis caused by the pandemic necessitated greater caution in monitoring patients post-discharge, due to the complexity and specificity of post-discharge care required by hospitalized COVID-19 patients. The majority of patients do not return to their pre-hospitalization health conditions at the time of discharge, often requiring specialized follow-up post-hospitalization^(4)^. Studies have identified functional decline and increased frailty in at least one-third of patients up to 3 months post-COVID-19 hospitalization.

Such conditions are associated with higher mortality and decreased quality of life for patients, as well as imposing greater responsibility on families, caregivers, and the healthcare system as a whole. These findings underscore the impact of the disease on patients and their families even after hospitalization, underscoring the importance of follow-up, personalized care plans, and rehabilitation^(5-6)^.

It is common for COVID-19 victims to be discharged home with gaps in care transition, resulting in an increased incidence of complications, readmissions, and lack of care follow-up. Factors such as social determinants, health conditions, and housing conditions influence the ability of patients and families to follow up with post-hospitalization care^(7)^.

Actions related to care transition aim to ensure the safe transfer of the patient between healthcare services, through care models that ensure continuity of care^(2)^. Care transition is an important strategy for implementing an integrated healthcare system, serving as a means to overcome care fragmentation and ensure continuity of care within the healthcare network^(8)^.

The nurse is the main professional responsible for facilitating successful transitions, ensuring that patients return home prepared and with adequate support^(9)^. Due to their experience and competence in teamwork, managing complex patients and their families, nurses serve as the primary link between professionals and services at different levels of care, often assuming the role of discharge planning, facilitating actions and interactions between professionals and services, patients and caregivers, with the aim of ensuring continuity of care post-hospital discharge^(3,10)^.

Among the primary benefits of effective care transitions are the reduction of readmissions and visits to the emergency department, particularly in patients with chronic diseases and advanced age. Decreasing mortality, hospital costs, adverse events, and increasing quality of life and patient satisfaction are also benefits already evidenced in studies^(3)^.

Various factors can influence the quality and experience of patient care transitions, including their expectations, level of knowledge, autonomy, emotional and physical well-being, as well as the care environment and level of transition planning^(2)^. Understanding patients’ and caregivers’ perceptions of the care transition process is essential for developing strategies that contribute to the development and implementation of more qualified transitions^(8)^ and becomes even more relevant due to the scarcity of international studies describing the quality of patient care transition after hospitalization for COVID-19.

In this regard, with the aim of assisting healthcare professionals and managers in identifying aspects to be improved in care transition, the question arises: how is the quality, from the perspective of patients and caregivers, of the care transition of COVID-19 patients evaluated from discharge from a University Hospital in southern Brazil?

## OBJECTIVE

To evaluate the quality of care transition from the hospital to the home of COVID-19 patients.

## METHODS

### Ethical considerations

The research adhered to the guidelines outlined in Resolution No. 466, dated December 12, 2012, of the National Health Council, which governs research involving human subjects. Approval for the study was granted by the Research Ethics Committee of the Federal University of Santa Catarina. As data collection was conducted via telephone calls, verbal consent was obtained at the time of the call, recorded, and securely archived under the researchers’ custody.

### Study design, timeframe, and location

This study is part of a multicenter research project titled “Evaluation of nursing care for patients with COVID-19 in Brazilian university hospitals,” funded by the Brazilian National Council for Scientific and Technological Development (CNPq), through public call MCTIC/CNPq/FNDCT/MS/SCTIE/Decit No. 07/2020 - Research for addressing COVID-19, its consequences, and other severe acute respiratory syndromes. The study adhered to the STROBE guidelines for reporting observational research.

Data collection took place from April to December 2021, a period marked by a significant rise in COVID-19 cases and deaths nationwide. The study was conducted at a University Hospital in Southern Brazil managed by the Brazilian Company of Hospital Services (EBSERH), selected as a regional healthcare reference. It is a large general hospital with 245 beds. Only inpatient units where professionals were involved in the care of adult patients with suspected or confirmed COVID-19 and discharged home were included in the study. Notably, the institution lacked specialized services to support the care transition process. Nurses and physicians primarily assumed responsibility for guiding patients and families, as well as providing documents such as discharge summaries and medication prescriptions.

### Population or sample

The study population comprised patients and caregivers discharged home after hospitalization for COVID-19. Inclusion criteria consisted of: age over 18 years, fluency in Brazilian Portuguese, hospitalization for a minimum of 72 hours for specific COVID-19 care, and hospital discharge exceeding seven days from the time of the telephone call.

The minimum sample size was calculated using the Winpepi program, version 11.65, based on the number of hospital beds, with a margin of error of 4 points, standard deviation of 17.1, and confidence level of 95%. This estimated a minimum sample of 63 patients.

### Study Protocol

Data collection was conducted through telephone calls, within a period of seven days after the patient’s discharge. Two instruments were utilized: 1) a characterization instrument; and 2) the Brazilian version of the Care Transitions Measure (CTM-15). The characterization instrument included variables such as age, gender, level of education, race, family income, total length of hospitalization, length of ICU stay, use of invasive mechanical ventilation, history of smoking, symptoms presented, and comorbidities.

The CTM-15, developed in the United States in 2002, aims to assess the quality of care transition between different healthcare services from the patient’s perspective. The instrument comprises 15 statements regarding the care transition process, categorized into 4 factors: Preparation for self-management; Understanding of medications; Preferences ensured; and Care plan. These statements are measured on a Likert scale with five response options ranging from “strongly agree” to “strongly disagree”^(8)^.

### Analysis of Results

The data were tabulated in an Excel spreadsheet and analyzed using the Statistical Package for the Social Sciences (SPSS) version 25. Categorical variables were presented using absolute and relative frequencies, while continuous variables were presented using measures of central tendency and dispersion.

To obtain the scores and analyze the quality of care transition, the sum of values for responses was calculated: Strongly disagree (1 point); Disagree (2 points); Agree (3 points); and Strongly agree (4 points). The option “I don’t know/don’t remember/does not apply” corresponds to 0 points and is analyzed separately, not included in the count.

The score, obtained by summing the values for responses, was divided by the number of questions answered with 1 to 4 points. This result was then transformed into a linear scale from zero to 100, following the authors’ instructions for the instrument^(11)^. The mean score across all participants was calculated, indicating the level of quality of care transition in that context.

Furthermore, the mean CTM-15 score was tested against independent variables using non-parametric tests such as Mann-Whitney, Kruskal-Wallis, and Spearman correlation. The significance level considered was 95% (p <0.05).

## RESULTS

A total of 78 patients who were discharged from the hospital after COVID-19 hospitalization participated in the study. In 54 (69.2%) interviews, the patients themselves responded to the instrument. Regarding sociodemographic characteristics, the majority of participants were male (57.7%), with a mean age of 53.7 years (SD 17.6), completed elementary education (43.7%), white race (74.6%), and with a family income between two and five minimum wages (62.5%). The length of hospitalization for patients varied from three to 105 days, with an average of 16.7 days (SD=16), and the length of ICU stay ranged from zero to 33 days (average 6 days; SD=8.9). The majority of patients did not require invasive mechanical ventilation. Regarding smoking history, less than half of the participants reported never having smoked. As for comorbidities, the most prevalent condition was Systemic Arterial Hypertension, followed by Obesity and Diabetes Mellitus. Most patients experienced fatigue, fever, dyspnea, arthralgia and myalgia, cough, and headache at least once during hospitalization (Table 1).

**Table 1:** Sociodemographic and Clinical Characteristics of Participants Included in the Study. Florianópolis, Santa Catarina, Brazil, 2022

Characteristics	n	%
Gender n=71		
Male	41	57.7
Female	30	42.3
Level of Education n=71		
Elementary School	31	43.7
High School	23	32.4
Higher Education	15	21.1
No Education	2	2.8
Race n=71		
White	53	74.6
Brown	12	16.9
Black	6	8.5
Other	-	-
Family Income n=64		
2 to 5 minimum wages	40	62.5
Up to 2 minimum wages	15	23.4
More than 5 minimum wages	8	12.5
No income	1	1.6
Use of Mechanical Ventilation n=70		
No	47	67.1
Yes	23	32.9
Smoking History n=70		
Former Smoker	34	48.6
Non-Smoker	33	47.1
Smoker	3	4.3
Comorbidities n=70		
Systemic Arterial Hypertension	34	48.6
Obesity	22	31.4
Diabetes Mellitus	19	27.1
Chronic Respiratory Disease	18	25.7
Cardiovascular Diseases	15	21.4
Renal Diseases	8	11.4
Cancer	7	10.0
Signs and Symptoms n=70		
Fatigue	67	95.7
Fever	59	84.3
Dyspnea	59	84.3
Myalgia and arthralgia	59	84.3
Cough	53	75.7
Headache	43	61.4
Diarrhea	35	50.0
Anosmia and ageusia	32	45.7
Nausea and vomiting	29	41.4

The total average score of CTM-15 was 70.8 (SD=17.0). The highest score (79.1) was attributed to item 4, “Received the information needed for self-care.” The items 6, related to understanding of warning signs and symptoms, and items 13 and 14, both related to patient understanding of medication therapy, followed suit. However, item 15, “Understanding of side effects,” had the lowest score (Table 2).

**Table 2:** Quality of care transition according to items of the Care Transitions Measure (CTM-15) instrument. Florianópolis, Santa Catarina, Brazil, 2022

Item		Factor	Mean	Standard Deviation
4	Received the information needed for self-care	1	79.1	24.4
6	Understands warning signs and symptoms	1	77.9	19.2
14	Understands how to take medications	2	77.5	18.3
13	Understands the reason for taking medications	2	77.5	19.1
9	Understands what is their responsibility	1	76.5	20.9
1	Agreed with the healthcare team on health goals and how they would be achieved	3	75.6	20.8
8	Understands what improves or worsens their health condition	1	75.6	21.3
5	Clearly understands how to take care of health	1	75.1	23.3
10	Feels confident they know what to do	1	71.5	22.9
2	Preferences considered to decide health needs	3	71.1	20.8
11	Feels confident they can do what is necessary	1	71.0	22.5
3	Preferences considered to decide where health needs are met	3	68.5	23.3
7	Received a written care plan	4	67.6	26.4
12	Received a written list of appointments or exams	4	62.3	25.5
15	Understands the side effects of medications	2	60.2	24.2

Among the factors of the CTM-15, we can observe that the highest score was attributed to Factor 1, ‘Preparation for self-management,’ and the lowest to Factor 4, ‘Care plan’ (Table 3).

**Table 3:** Quality of care transition according to the factors of the Care Transitions Measure (CTM-15) instrument. Florianópolis, Santa Catarina, Brazil, 2022

Factor		Mean	Standard Deviation
1	Preparation for self-management	74.1	19.4
2	Understanding of medications	70.8	17.3
3	Ensured preferences	69.6	21.3
4	Care plan	60.2	25.1

The quality of care transition was correlated with the sociodemographic characteristics of the study participants. In this analysis, a relationship was highlighted concerning race, where self-declared mixed-race participants attributed lower scores to the quality of care transition, especially regarding Factor 3 of the CTM-15, related to ensured preferences (Table 4).

**Table 4:** Quality of care transition according to the Factors of the Care Transitions Measure (CTM-15) instrument and the sociodemographic characteristics of the participants. Florianópolis, Santa Catarina, Brazil, 2022

	Factor 1	Factor 2	Factor 3	Factor 4	Total
	**Mean (SD)**	**Mean (SD)**	**Mean (SD)**	**Mean (SD)**	**Mean (SD)**
Gender					
Female	75.1 (22.1)	70.9 (18.2)	71.9 (22.4)	60.0 (26.5)	71.5 (18.7)
Male	72.1 (18.1)	69.5 (17.1)	66.5 (20.8)	58.3 (24.5)	68.9 (15.8)
*p* value	0.446	0.622	0.266	0.822	0.533
Level of Education					
No education	66.7 -	66.7 -	66.7 -	66.7 -	66.7 -
Elementary School	77.3 (16.8)	69.6(18.7)	70.6(22.1)	60.2 (24.2)	72.2 (16.2)
High School	70.4 (23.6)	73.2 (18.4)	65.7 (19.5)	62.9 (25.7)	69.2 (18.8)
Higher Education	70.8 (20.4)	66.7 (14.6)	70.0 (25.2)	50.0 (27.5)	67.2 (17.7)
*p* value	0.419	0.537	0.894	0.468	0.750
Race					
White	73.9 (21.4)	71.2 (18.2)	70.6 (22.6)	59.0 (28.5)	70.9 (18.4)
Black	77.0 (17.9)	70.4 (15.2)	75.9 (19.1)	58.3 (13.9)	72.8 (13.4)
Mixed Race	69.1 (12.6)	64.7 (14.8)	57.4 (13.3)	59.7 (11.1)	64.5 (10.7)
*p* value	0.684	0.505	0.047	0.960	0.443
Family Income					
Up to 2 MW^*^	65.4 (20.7)	68.9 (16.9)	63.0 (16.6)	58.9 (18.8)	64.9 (16.0)
2 to 5 MW ^*^	77.1 (16.7)	70.7 (18.5)	70.3 (22.0)	63.3 (28.4)	72.8 (16.6)
More than 5 MW ^*^	78.6 (21.6)	75.0 (19.5)	79.2 (21.0)	47.9 (24.3)	73.9 (18.9)
No income	100 -	55.6 -	100 -	66.7 -	86.7 -
*p* value	0.157	0.489	0.128	0.514	0.424

* *MW= minimum wages*

In relation to the clinical characteristics of the participants, it is noted that a higher score was attributed by participants who presented symptoms of dyspnea, cough, anosmia, and ageusia during the COVID-19 infection (Table 5).

**Table 5:** Quality of care transition according to the Factors of the Care Transitions Measure (CTM-15) instrument and the clinical characteristics of the participants. Florianópolis, Santa Catarina, Brazil, 2022

	Factor 1	Factor 2	Factor 3	Factor 4	Total
	Mean (SD)	Mean (SD)	Mean (SD)	Mean (SD)	Mean (SD)
Mechanical ventilation use					
No	71.6 (20.1)	68.9 (16.9)	65.6 (22.6)	55.8 (26.6)	68.0 (17.2)
Yes	76.8 (17.3)	72.5 (18.6)	76.8 (16.0)	65.9 (21.6)	74.5 (14.7)
*p* value	0.427	0.314	0.047	0.079	0.116
Smoking history					
Non-smoker	71.0 (21.2)	69.4 (18.2)	72.7 (18.0)	59.6 (28.0)	69.5 (17.2)
Smoker	73.0 (16.7)	66.7 (11.1)	66.7 (11.1)	44.4 (34.7)	67.3 (14.9)
Former smoker	75.6 (19.2)	71.1 (17.4)	66.2 (24.4)	60.1 (22.0)	71.0 (17.6)
*p* value	0.569	0.970	0.539	0.578	0.758
Chronic respiratory disease					
No	73.7 (20.8)	70.0 (17.9)	68.7 (22.1)	57.5 (26.1)	70.0 (17.4)
Yes	72.2 (17.8)	70.4 (16.6)	71.0 (18.7)	63.9 (23.0)	77.6 (16.5)
*p* value	0.588	0.972	0.917	0.462	0.840
Systemic Arterial Hypertension					
No	72.4 (22.2)	70.1 (18.9)	67.6 (23.4)	60.2 (24.3)	69.5 (19.2)
Yes	74.4 (17.5)	70.1 (16.0)	71.1 (18.8)	58.1 (26.7)	70.8 (14.8)
*p* value	0.762	0.520	0.526	0.768	0.851
Cardiovascular diseases					
No	75.0 (19.9)	72.0 (18.1)	70.2 (21.2)	61.7 (25.4)	71.8 (17.1)
Yes	67.3 (19.5)	63.0 (13.1)	65.9 (21.6)	50.0 (23.6)	64.1 (16.2)
*p* value	0.087	0.112	0.444	0.088	0.088
Diabetes Mellitus					
No	72.6 (19.4)	68.4 (16.7)	69.7 (18.7)	56.9 (25.6)	68.2 (16.1)
Yes	75.4 (21.6)	74.6 (19.0)	68.1 (27.3)	65.7 (23.9)	72.7 (19.8)
*p* value	0.622	0.118	0.734	0.269	0.358
Renal diseases					
No	74.4 (18.8)	69.8 (17.5)	71.9 (18.0)	61.5 (25.2)	71.3 (16.2)
Yes	65.5 (27.2)	72.2 (17.8)	49.3 (33.3)	41.7 (19.9)	61.1 (22.3)
*p* value	0.264	0.856	0.063	0.038	0.142
Obesity					
No	73.9 (21.0)	71.4 (17.5)	69.1 (22.9)	61.0 (23.6)	70.9 (17.6)
Yes	72.1 (17.8)	67.2 (17.3)	69.7 (17.6)	55.3 (28.8)	68.5 (16.1)
*p* value	0.853	0.340	0.933	0.259	0.539
Cancer					
No	74.0 (19.1)	69.6 (17.5)	70.2 (19.8)	59.4 (24.6)	70.6 (16.3)
Yes	67.4 (27.1)	74.6 (17.8)	61.1 (31.9)	57.1 (33.1)	66.3 (24.3)
*p* value	0.515	0.418	0.409	0.676	0.543
Fever					
No	73.6 (21.3)	68.7 (14.8)	65.7 (23.0)	57.6 (20.2)	69.0 (17.9)
Yes	73.3 (19.8)	70.3 (18.0)	70.0 (21.0)	59.5 (26.3)	70.4 (17.1)
*p* value	0.961	0.987	0.523	0.687	0.710
Fatigue					
No	41.3 (24.0)	66.7 -	59.3 (12.8)	44.4 (19.3)	50.4 (15.6)
Yes	74.8 (18.7)	70.2 (17.8)	69.7 (21.5)	59.9 (25.5)	71.0 (16.7)
*p* value	0.019	0.978	0.326	0.271	0.067
Dyspnea					
No	58.9 (23.5)	64.7 (15.6)	63.1 (24.4)	43.9 (17.1)	59.1 (17.6)
Yes	76.0 (18.2)	71.1 (17.7)	70.4 (20.6)	62.1 (25.7)	72.2 (16.3)
*p* value	0.025	0.473	0.249	0.018	0.032
Cough					
No	63.0 (22.3)	67.3 (13.3)	54.6 (24.9)	54.9 (19.3)	61.2 (18.0)
Yes	76.6 (18.1)	71.0 (18.6)	74.0 (17.7)	60.6 (27.0)	73.0 (16.0)
*p* value	0.040	0.646	0.003	0.336	0.024
Anosmia and ageusia					
No	68.7 (18.7)	66.7 (14.6)	64.8 (19.1)	57.0 (22.1)	66.2 (15.3)
Yes	78.9 (20.1)	74.1 (19.7)	74.7 (22.6)	61.8 (28.9)	74.8 (18.2)
*p* value	0.027	0.073	0.024	0.545	0.030
Headache					
No	71.4 (19.5)	70.0 (17.1)	65.2 (24.0)	59.9 (20.8)	68.5 (17.2)
Yes	74.5 (20.3)	70.2 (17.8)	71.8 (19.2)	58.7 (28.1)	71.2 (17.2)
*p* value	0.563	0.896	0.340	0.880	0.566
Myalgia and arthralgia					
No	64.5 (20.0)	66.7 (17.9)	67.7 (16.8)	54.6 (23.7)	64.2 (16.6)
Yes	75.0 (19.6)	70.7 (17.4)	69.6 (22.0)	60.1 (25.7)	71.2 (17.1)
*p* value	0.145	0.438	0.578	0.440	0.180
Nausea and vomiting					
No	74.1 (18.2)	71.0 (17.9)	70.9 (20.5)	58.9 (25.9)	71.0 (16.5)
Yes	72.3 (22.4)	68.8 (17.0)	67.1 (22.3)	59.5 (25.0)	68.9 (18.1)
*p* value	0.805	0.644	0.384	0.703	0.694
Diarrhea					
No	69.5 (18.5)	66.7 (18.9)	68.6 (20.3)	56.7 (24.0)	67.1 (16.4)
Yes	77.1 (20.9)	73.5 (15.4)	70.0 (22.4)	61.8 (26.8)	73.1 (17.5)
*p* value	0.081	0.065	0.550	0.305	0.115

A lower score was observed in Factor 1, “Preparation for self-management,” among patients who reported fatigue compared to those who denied this symptom (41.3 vs. 74.8, respectively) and other groups. The lowest score in Factor 2, “Understanding of medications,” was attributed to participants with cardiovascular diseases (63.0). Factors 3, “Preferences ensured,” and 4, “Care plan,” obtained significantly lower scores among patients with kidney diseases (49.3 and 41.7, respectively) compared to those without (71.9 and 61.5, respectively), with a statistically significant difference in the assignment of scores in Factor 4 in patients with or without this comorbidity (p=0.038). There is also a statistically significant difference in scores in Factor 1 and 3 (p=0.040 and p=0.003) for patients who did or did not present cough as a symptom and in Factor 1 scores between patients who did or did not have anosmia and ageusia (p=0.027).

## DISCUSSION

The findings of this study facilitated the assessment of care transition among patients hospitalized for COVID-19. The mean CTM-15 score in this study was 70.8 (SD 17.0), signifying a moderate quality of care transition. When interpreting the instrument, a higher mean score indicates a positive perception of care transition by patients. While there is no defined cutoff point for CTM-15 scores in the literature, studies conducted with adult patients in Brazil and the United States have reported scores ranging from 69.5 to 78.5^(8,12-14)^. Given the pandemic context of hospital overcrowding, healthcare professional overload, and resource scarcity, a moderate quality of care transition is deemed a favorable outcome.

Due to the current nature of the disease, limited studies describing the care transition experience of COVID-19 hospitalized patients have been identified. A study conducted in Spain on the experiences of COVID-19 hospitalized patients revealed that, despite expressing gratitude and positivity about the care received, many patients felt they did not consistently receive the expected level of care due to shortages in material and human resources caused by the pandemic^(15)^. Another study conducted in Canada showed that the majority of patients perceived their discharge as rushed and felt ill-prepared and inadequately informed upon returning home. Some reported not receiving essential information and guidance at discharge, such as support contacts, counseling, and a care plan, often leading to returns to the hospital emergency department. Patients also linked these gaps in the transition process and overall care to the pandemic’s conditions and the prevailing restriction protocols^(16)^.

The highest score in the study was recorded for Factor 1, “Preparation for self-management,” particularly for item 4, “Had the information needed for self-care,” indicating effective sharing and comprehension of information during hospitalization, along with patients’ confidence in self-managing their health needs at home. This finding is encouraging, as patient readiness for self-management of health conditions and home-based self-care stands among the most impactful interventions for establishing well-executed care transitions^(17)^.

The second-highest score in the study was attributed to Factor 2, Understanding of medications. However, item 15, “Understands the side effects of medications,” received the lowest score, revealing a gap in the guidance provided at discharge. The findings suggest that while information about medications is provided, patients are not adequately informed about potential side effects, receiving only details such as dosage, timing, and administration route^(12)^.

Guidance on medication use that includes information about side effects is crucial to prevent adverse events after hospital discharge^(3)^. Medication prescriptions made for hospital discharge present a significant potential problem, especially due to the discrepancy between medication therapy before and after hospitalization, as only 10% of patients are discharged with the same medication therapy as admission^(18)^. Medication reconciliation actions can yield positive outcomes in this regard. Studies have shown a decrease in the percentage of rehospitalizations and a reduction in adverse events in patients who received this type of intervention, which can also be performed by nurses^(3,19)^.

Factor 4, Care plan, had the lowest score in the study. The scores of items 7 “Received a written care plan” and 12 “Received a written list of appointments or exams” indicate that many patients were discharged without receiving a structured care plan and referrals for post-discharge follow-up. This finding is similar to those described in the literature, demonstrating a common fragility in different health services, exacerbated by the pandemic context. It is noteworthy that planning and building a care plan are essential to ensure patient preparedness for self-management at home, while referrals for post-hospital follow-up facilitate the patient’s responsible integration into different health services, providing continuity of care^(12-13)^.

Outpatient follow-up allows for rigorous monitoring and symptom management after discharge, enabling problems and concerns to be detected and addressed early, thus avoiding unnecessary rehospitalizations. Communication between different levels of care aids in the implementation of best transition practices and ensures continuity of care. However, in Brazil, there is a perception of fragmentation and lack of coordination between hospitals and other levels of care^(9,19)^.

The nurse is usually responsible for care coordination, identifying needs and preferences, and developing individualized care plans. This practice occurs more delimitedly in countries where there are nurses specifically directed to hospital discharge coordination and planning activities, called liaison nurses. These professionals play a fundamental role in continuity of care and are responsible for discharge planning, health education, identification of strengths and weaknesses, and transfer of patient information between the hospital and other health services^(20)^.

The study also revealed that patients experiencing symptoms such as dyspnea, cough, and anosmia/ageusia tended to assign higher scores compared to those without these symptoms, particularly concerning Factor 1, which relates to self-management actions for the disease. It’s important to note that these symptoms have been consistently associated with COVID-19 complications since the onset of the disease. Therefore, these findings may indicate that these symptoms served as warning signs of worsening clinical conditions, requiring increased attention from healthcare professionals during the care transition^(21)^.

Regarding patient characteristics, lower scores were observed among participants of mixed race and with lower family income. In a study conducted in the United States, Black patients reported fewer follow-up appointments, less likelihood of receiving contact to address concerns, and less provision of equipment for continued home care. According to the authors, such disparities may be attributed to factors such as limited access to healthcare and health literacy, which are also linked to financial constraints^(22)^. However, caution should be exercised in interpreting the results of this study, as despite differences in CTM-15 scores, the comparison between categories did not reach statistical significance, necessitating further studies involving larger populations to gain a deeper understanding of the phenomenon.

The care transition process, as a whole, is complex and involves various issues related to the performance of professionals, the utilization of protocols, among others. Therefore, evaluating care transition within a context presents a challenge that requires a multifaceted approach encompassing multiple perspectives, instruments, and indicators^(12)^.

### Limitations of the Study

This study has some limitations that need to be considered when interpreting its results. Firstly, the sample size may restrict the generalization of findings to other populations or care contexts. While the participants represent a significant sample, studies with larger samples could offer a more comprehensive and robust insight into the quality of care transition in patients with COVID-19.

Additionally, the retrospective nature of the study and data collection through telephone interviews may introduce memory or response biases in participants, potentially affecting the accuracy and reliability of the collected data.

### Contributions to the Nursing Field

This study makes significant contributions to the nursing field by highlighting essential aspects of care transition in patients hospitalized for COVID-19. By identifying key strengths and weaknesses in the quality of care transition, nurses can target specific interventions to enhance patients’ experience and outcomes during this critical phase.

The findings emphasize the importance of providing clear and comprehensive information to patients about self-care, medication, and warning signs, with the aim of empowering patients to take an active role in their recovery after hospital discharge.

## CONCLUSIONS

It was observed that the quality of care transition for patients hospitalized with COVID-19 at a University Hospital was moderate. Aspects related to the guidance received during hospitalization, patients’ understanding of this guidance, and the incorporation of patients’ preferences into post-discharge care showed higher scores. However, the main weaknesses observed were related to understanding the side effects of medications, care planning, and post-discharge referrals. In conclusion, further research is needed to better assess care transitions, exploring different perspectives, social and health determinants, and seeking strategies to improve processes and actions in healthcare institutions.

## Supplementary Material





## Data Availability

https://doi.org/10.48331/scielodata.B4L4S8
