# Supplementary material for: Quality of Care Transition for COVID-19 Patients in a University Hospital in Southern Brazil
Source: Rev Bras Enferm. 2024 Jun 28;77(Suppl 1):e20230402. doi: 10.1590/0034-7167-2023-0402 (PMC11213544; doi:10.1590/0034-7167-2023-0402)
Supplement: Supplementary file 1 [file 0034-7167-reben-77-s1-e20230402-suppl1.pdf]

● Título do conjunto de dados:

- banco de dados da pesquisa

● Informações de contato (nome, instituição e e-mail) do pesquisador correspondente / principal ou da pessoa responsável pela coleta dos dados:

- Caroline Cechinel Peiter, Universidade Federal de Santa Catarina, carolcechinel@gmail.com

● Data de coleta dos dados (data única ou intervalo de tempo):

- abril e dezembro de 2021

● Visão geral dos dados e arquivos (breve descrição dos dados que cada arquivo contém, data de criação de cada arquivo e como eles se relacionam entre si, etc)

- Trata-se de um único arquivo, em arquivo do Microsoft Excel, contendo a planilha utilizada para organização dos dados dos participantes incluídos no estudo.

● Descrição dos métodos de coleta ou geração dos dados;

- Estudo quantitativo do tipo transversal, realizado em um Hospital Universitário ao Sul do Brasil. Participaram 78 pacientes que tiveram alta hospitalar após internação por Covid-19. A coleta de dados foi realizada por telefone utilizando um instrumento de caracterização e a versão brasileira do instrumento Care Transitions Measure (CTM-15).

● Descrição dos métodos usados para o processamento dos dados;

- Os dados foram analisados por meio de estatística descritiva e analítica. Os dados foram organizados e processados no programa Statistical Package for the Social Science (SPSS)versão 25, sendo aplicados os testes Mann-Whitney, Kruskal-Wallis e correlação de Spearman. O nível de significância considerado foi de 95% ($p<0,05$).

● Informações específicas dos dados (lista de variáveis, unidades de medida, definições de códigos ou símbolos, calibragem de equipamentos, etc).

Variáveis contínuas:
- Qualidade da transição do cuidado: 1 a 100 pontos
- Fator 1 - Preparação para autogerenciamento: 1 a 100 pontos
- Fator 2 - Entendimento sobre medicações: 1 a 100 pontos
- Fator 3 - Preferências asseguradas: 1 a 100 pontos
- Fator 4 - Plano de cuidado: 1 a 100 pontos
- idade: anos completos
- tempo total de internação: dias
- tempo de internação em UTI: dias

Variáveis categóricas:
- gênero: masculino; feminino
- grau de instrução: sem instrução; ensino fundamental; ensino médio; ensino superior

- raça: branca; parda; preta; indígena; amarela
- renda familiar: sem renda; até dois salários mínimos; dois a cinco salários mínimos; mais de cinco salários mínimos
- uso de ventilação mecânica invasiva: não; sim
- histórico de tabagismo: não fumante, fumante; ex fumante
- sintomas apresentados: fadiga; febre; dispneia; mialgia e artralgia; tosse; cefaleia; diarreia; anosmia e ageusia; náuseas e vômitos
- comorbidades: hipertensão arterial sistêmica; obesidade; diabetes; doença respiratória crônica; doenças cardiovasculares; doenças renais; câncer
