# Supplementary material for: Quality of Care Transition for COVID-19 Patients in a University Hospital in Southern Brazil
Source: Rev Bras Enferm. 2024 Jun 28;77(Suppl 1):e20230402. doi: 10.1590/0034-7167-2023-0402 (PMC11213544; doi:10.1590/0034-7167-2023-0402)
Supplement: Supplementary file 2 [file 0034-7167-reben-77-s1-e20230402-suppl2.pdf]

Número de	As primeira	2. A equipe	3. A equipe	As próxima	5. Quando	6. Quando	7. Quando	8. Quando	9. Quando
A906	2 - Discord	2 - Discord	3 - Concor	4 - Concor	3 - Concor	3 - Concor	4 - Concor	4 - Concor	3 - Concor
A579	3 - Concor	3 - Concor	3 - Concor	2 - Discord	3 - Concor	2 - Discord	2 - Discord	2 - Discord	3 - Concor
A562	3 - Concor	3 - Concor	3 - Concor	4 - Concor	3 - Concor	3 - Concor	2 - Discord	3 - Concor	3 - Concor
A534	3 - Concor	2 - Discord	1 - Discord	2 - Discord	2 - Discord	3 - Concor	0 - Não sei	3 - Concor	3 - Concor
A586	1 - Discord	3 - Concor	3 - Concor	1 - Discord	1 - Discord	3 - Concor	1 - Discord	2 - Discord	2 - Discord
A904	3 - Concor	2 - Discord	2 - Discord	2 - Discord	2 - Discord	2 - Discord	2 - Discord	3 - Concor	3 - Concor
A602	3 - Concor	4 - Concor	4 - Concor	4 - Concor	4 - Concor	4 - Concor	4 - Concor	4 - Concor	4 - Concor
A486	4 - Concor	4 - Concor	0 - Não sei	4 - Concor	4 - Concor	4 - Concor	4 - Concor	4 - Concor	4 - Concor
A532	3 - Concor	3 - Concor	3 - Concor	4 - Concor	4 - Concor	4 - Concor	3 - Concor	4 - Concor	4 - Concor
A494	3 - Concor	4 - Concor	4 - Concor	4 - Concor	4 - Concor	4 - Concor	4 - Concor	4 - Concor	4 - Concor
A611	2 - Discord	3 - Concor	3 - Concor	3 - Concor	3 - Concor	3 - Concor	3 - Concor	3 - Concor	4 - Concor
A182	4 - Concor	4 - Concor	4 - Concor	4 - Concor	4 - Concor	3 - Concor	2 - Discord	4 - Concor	4 - Concor
A671	3 - Concor	4 - Concor	4 - Concor	3 - Concor	3 - Concor	3 - Concor	4 - Concor	3 - Concor	4 - Concor
A499	2 - Discord	2 - Discord	2 - Discord	3 - Concor	3 - Concor	4 - Concor	3 - Concor	3 - Concor	3 - Concor
A453	3 - Concor	3 - Concor	3 - Concor	3 - Concor	3 - Concor	3 - Concor	3 - Concor	3 - Concor	3 - Concor
A889	3 - Concor	3 - Concor	2 - Discord	3 - Concor	3 - Concor	3 - Concor	3 - Concor	3 - Concor	3 - Concor
A914	2 - Discord	2 - Discord	2 - Discord	2 - Discord	2 - Discord	3 - Concor	2 - Discord	2 - Discord	3 - Concor
A644	3 - Concor	3 - Concor	3 - Concor	1 - Discord	2 - Discord	3 - Concor	2 - Discord	2 - Discord	2 - Discord
A485	4 - Concor	4 - Concor	4 - Concor	4 - Concor	4 - Concor	4 - Concor	4 - Concor	4 - Concor	4 - Concor
A830	0 - Não sei	1 - Discord	2 - Discord	3 - Concor	2 - Discord	3 - Concor	2 - Discord	2 - Discord	3 - Concor
A869	3 - Concor	3 - Concor	3 - Concor	3 - Concor	3 - Concor	3 - Concor	3 - Concor	3 - Concor	3 - Concor
A000	3 - Concor	3 - Concor	4 - Concor	4 - Concor	4 - Concor	4 - Concor	4 - Concor	4 - Concor	4 - Concor
A090	3 - Concor	3 - Concor	3 - Concor	4 - Concor	4 - Concor	4 - Concor	2 - Discord	4 - Concor	4 - Concor
A877	4 - Concor	3 - Concor	3 - Concor	4 - Concor	4 - Concor	4 - Concor	4 - Concor	4 - Concor	4 - Concor
A897	2 - Discord	2 - Discord	2 - Discord	3 - Concor	2 - Discord	3 - Concor	2 - Discord	3 - Concor	2 - Discord
A573	3 - Concor	3 - Concor	3 - Concor	3 - Concor	3 - Concor	3 - Concor	3 - Concor	3 - Concor	3 - Concor
A749	4 - Concor	4 - Concor	4 - Concor	4 - Concor	4 - Concor	4 - Concor	3 - Concor	4 - Concor	4 - Concor
A920	3 - Concor	3 - Concor	3 - Concor	4 - Concor	4 - Concor	3 - Concor	2 - Discord	3 - Concor	3 - Concor
A574	4 - Concor	3 - Concor	3 - Concor	4 - Concor	4 - Concor	4 - Concor	4 - Concor	4 - Concor	4 - Concor
A876	4 - Concor	3 - Concor	3 - Concor	4 - Concor	4 - Concor	4 - Concor	4 - Concor	4 - Concor	3 - Concor
A542	3 - Concor	3 - Concor	3 - Concor	4 - Concor	3 - Concor	3 - Concor	3 - Concor	3 - Concor	3 - Concor
A653	3 - Concor	4 - Concor	4 - Concor	4 - Concor	4 - Concor	4 - Concor	4 - Concor	4 - Concor	4 - Concor
A066	2 - Discord	2 - Discord	2 - Discord	2 - Discord	2 - Discord	3 - Concor	2 - Discord	2 - Discord	2 - Discord
A559	3 - Concor	3 - Concor	3 - Concor	4 - Concor	4 - Concor	3 - Concor	3 - Concor	4 - Concor	3 - Concor
A609	3 - Concor	3 - Concor	3 - Concor	4 - Concor	0 - Não sei	4 - Concor	0 - Não sei	4 - Concor	4 - Concor
A912	3 - Concor	3 - Concor	3 - Concor	2 - Discord	2 - Discord	3 - Concor	2 - Discord	2 - Discord	2 - Discord
A204	4 - Concor	4 - Concor	4 - Concor	4 - Concor	4 - Concor	4 - Concor	4 - Concor	4 - Concor	4 - Concor
A513	4 - Concor	0 - Não sei	4 - Concor	4 - Concor	3 - Concor	4 - Concor	4 - Concor	3 - Concor	4 - Concor
A564	3 - Concor	3 - Concor	3 - Concor	4 - Concor	3 - Concor	3 - Concor	3 - Concor	3 - Concor	3 - Concor
A583	4 - Concor	2 - Discord	1 - Discord	4 - Concor	4 - Concor	3 - Concor	4 - Concor	4 - Concor	4 - Concor
A136	3 - Concor	3 - Concor	3 - Concor	3 - Concor	3 - Concor	3 - Concor	3 - Concor	3 - Concor	3 - Concor
A496	3 - Concor	3 - Concor	3 - Concor	3 - Concor	3 - Concor	3 - Concor	3 - Concor	3 - Concor	3 - Concor
A662	4 - Concor	4 - Concor	4 - Concor	4 - Concor	4 - Concor	4 - Concor	4 - Concor	4 - Concor	3 - Concor
A091	4 - Concor	4 - Concor	2 - Discord	3 - Concor	3 - Concor	4 - Concor	1 - Discord	3 - Concor	3 - Concor
A866	3 - Concor	3 - Concor	3 - Concor	3 - Concor	3 - Concor	3 - Concor	3 - Concor	3 - Concor	3 - Concor
A626	4 - Concor	4 - Concor	4 - Concor	4 - Concor	4 - Concor	4 - Concor	2 - Discord	4 - Concor	4 - Concor
A867	4 - Concor	2 - Discord	2 - Discord	4 - Concor	4 - Concor	4 - Concor	4 - Concor	4 - Concor	4 - Concor
A873	4 - Concor	4 - Concor	3 - Concor	3 - Concor	3 - Concor	4 - Concor	0 - Não sei	3 - Concor	3 - Concor
A575	3 - Concor	3 - Concor	3 - Concor	3 - Concor	3 - Concor	3 - Concor	3 - Concor	3 - Concor	3 - Concor
A138	3 - Concor	3 - Concor	3 - Concor	3 - Concor	3 - Concor	3 - Concor	3 - Concor	3 - Concor	3 - Concor
A911	3 - Concor	3 - Concor	3 - Concor	3 - Concor	3 - Concor	3 - Concor	0 - Não sei	3 - Concor	2 - Discord
A701	2 - Discord	3 - Concor	3 - Concor	3 - Concor	3 - Concor	3 - Concor	2 - Discord	3 - Concor	3 - Concor
A684	3 - Concor	3 - Concor	3 - Concor	2 - Discord	2 - Discord	3 - Concor	3 - Concor	3 - Concor	4 - Concor

A474	3 - Concor	3 - Concor	2 - Discord	2 - Discord	3 - Concor	3 - Concor	2 - Discord	3 - Concor	3 - Concor
A546	3 - Concor	3 - Concor	2 - Discord	3 - Concor	3 - Concor	3 - Concor	3 - Concor	3 - Concor	3 - Concor
A464	4 - Concor	4 - Concor	4 - Concor	4 - Concor	4 - Concor	4 - Concor	2 - Discord	3 - Concor	3 - Concor
A473	3 - Concor	3 - Concor	3 - Concor	4 - Concor	3 - Concor	4 - Concor	3 - Concor	4 - Concor	3 - Concor
A527	4 - Concor	4 - Concor	4 - Concor	3 - Concor	4 - Concor	4 - Concor	4 - Concor	4 - Concor	4 - Concor
A882	3 - Concor	3 - Concor	4 - Concor	4 - Concor	3 - Concor	3 - Concor	3 - Concor	3 - Concor	3 - Concor
A549	4 - Concor	3 - Concor	3 - Concor	4 - Concor	3 - Concor	3 - Concor	2 - Discord	4 - Concor	3 - Concor
A724	3 - Concor	2 - Discord	2 - Discord	2 - Discord	1 - Discord	1 - Discord	1 - Discord	3 - Concor	3 - Concor
A511	4 - Concor	4 - Concor	3 - Concor	4 - Concor	4 - Concor	3 - Concor	3 - Concor	4 - Concor	4 - Concor
A192	3 - Concor	3 - Concor	3 - Concor	3 - Concor	3 - Concor	3 - Concor	3 - Concor	3 - Concor	3 - Concor
A524	3 - Concor	3 - Concor	4 - Concor	4 - Concor	3 - Concor	3 - Concor	3 - Concor	3 - Concor	3 - Concor
A172	4 - Concor	4 - Concor	4 - Concor	4 - Concor	4 - Concor	4 - Concor	4 - Concor	4 - Concor	4 - Concor
A121	3 - Concor	3 - Concor	2 - Discord	3 - Concor	4 - Concor	3 - Concor	2 - Discord	3 - Concor	3 - Concor
A607	3 - Concor	3 - Concor	3 - Concor	3 - Concor	3 - Concor	3 - Concor	3 - Concor	3 - Concor	3 - Concor
A591	4 - Concor	4 - Concor	4 - Concor	4 - Concor	4 - Concor	4 - Concor	4 - Concor	4 - Concor	4 - Concor
A896	1 - Discord	1 - Discord	1 - Discord	1 - Discord	2 - Discord	2 - Discord	2 - Discord	2 - Discord	3 - Concor
A523	4 - Concor	3 - Concor	3 - Concor	4 - Concor	4 - Concor	4 - Concor	4 - Concor	4 - Concor	4 - Concor
A637	4 - Concor	3 - Concor	2 - Discord	4 - Concor	3 - Concor	3 - Concor	4 - Concor	3 - Concor	3 - Concor
A623	3 - Concor	3 - Concor	3 - Concor	3 - Concor	3 - Concor	3 - Concor	3 - Concor	3 - Concor	3 - Concor
A854	3 - Concor	3 - Concor	2 - Discord	2 - Discord	2 - Discord	3 - Concor	3 - Concor	3 - Concor	3 - Concor
A696	4 - Concor	4 - Concor	4 - Concor	4 - Concor	4 - Concor	4 - Concor	4 - Concor	3 - Concor	4 - Concor
A762	4 - Concor	3 - Concor	3 - Concor	4 - Concor	4 - Concor	3 - Concor	3 - Concor	3 - Concor	4 - Concor
A751	4 - Concor	4 - Concor	3 - Concor	3 - Concor	4 - Concor	4 - Concor	4 - Concor	4 - Concor	4 - Concor
A895	4 - Concor	3 - Concor	4 - Concor	2 - Discord	3 - Concor	4 - Concor	2 - Discord	3 - Concor	2 - Discord
A909	3 - Concor	3 - Concor	2 - Discord	4 - Concor	3 - Concor	2 - Discord	2 - Discord	3 - Concor	4 - Concor

10. Quand	11. Quand	A próxima	As próxima	14. Quand	15. Quand	Quem resp	Vínculo co	Qual é a su	Eu vou lista
3 - Concor	3 - Concor	1 - Discord	2 - Discord	3 - Concor	2 - Discord	Paciente	Não se apl	37	2 - Femini
2 - Discord	2 - Discord	2 - Discord	3 - Concor	3 - Concor	2 - Discord	Cuidador	Familiar	57	1 - Masculi
3 - Concor	3 - Concor	2 - Discord	3 - Concor	3 - Concor	3 - Concor	Paciente	Não se apl	26	1 - Masculi
3 - Concor	4 - Concor	1 - Discord	2 - Discord	4 - Concor	2 - Discord	Paciente	Não se apl	52	1 - Masculi
1 - Discord	1 - Discord	3 - Concor	3 - Concor	4 - Concor	2 - Discord	Paciente	Não se apl	34	2 - Femini
3 - Concor	2 - Discord	3 - Concor	3 - Concor	3 - Concor	1 - Discord	Paciente	Não se apl	21	2 - Femini
4 - Concor	4 - Concor	4 - Concor	4 - Concor	4 - Concor	4 - Concor	Paciente	Não se apl	24	2 - Femini
4 - Concor	4 - Concor	1 - Discord	4 - Concor	4 - Concor	1 - Discord	Paciente	Profissiona	55	2 - Femini
3 - Concor	4 - Concor	3 - Concor	4 - Concor	4 - Concor	4 - Concor	Paciente	Não se apl	50	1 - Masculi
4 - Concor	4 - Concor	3 - Concor	4 - Concor	4 - Concor	3 - Concor	Paciente	Profissiona	23	1 - Masculi
3 - Concor	3 - Concor	2 - Discord	3 - Concor	3 - Concor	2 - Discord	Paciente	Não se apl	47	1 - Masculi
3 - Concor	2 - Discord	1 - Discord	4 - Concor	3 - Concor	2 - Discord	Paciente	Não se apl	52	1 - Masculi
4 - Concor	4 - Concor	3 - Concor	4 - Concor	4 - Concor	4 - Concor	Cuidador	Familiar	59	1 - Masculi
3 - Concor	4 - Concor	2 - Discord	0 - Não sei	0 - Não sei	0 - Não sei	Paciente	Profissiona	57	2 - Femini
3 - Concor	3 - Concor	3 - Concor	3 - Concor	3 - Concor	3 - Concor	Paciente	Não se apl	64	1 - Masculi
3 - Concor	3 - Concor	2 - Discord	3 - Concor	3 - Concor	3 - Concor	Paciente	Não se apl	99 - Entrev	99 - Entrev
2 - Discord	3 - Concor	2 - Discord	3 - Concor	3 - Concor	2 - Discord	Paciente	Não se apl	38	2 - Femini
2 - Discord	3 - Concor	3 - Concor	3 - Concor	3 - Concor	2 - Discord	Paciente	Não se apl	36	1 - Masculi
4 - Concor	4 - Concor	1 - Discord	4 - Concor	4 - Concor	4 - Concor	Cuidador	Profissiona	64	1 - Masculi
2 - Discord	2 - Discord	2 - Discord	3 - Concor	3 - Concor	3 - Concor	Cuidador	Familiar	67	1 - Masculi
3 - Concor	3 - Concor	3 - Concor	3 - Concor	3 - Concor	2 - Discord	Cuidador	Familiar	37	1 - Masculi
4 - Concor	4 - Concor	4 - Concor	4 - Concor	4 - Concor	4 - Concor	Paciente	Não se apl	62	1 - Masculi
4 - Concor	4 - Concor	3 - Concor	4 - Concor	4 - Concor	4 - Concor	Paciente	Não se apl	56	2 - Femini
3 - Concor	3 - Concor	4 - Concor	4 - Concor	4 - Concor	3 - Concor	Paciente	Não se apl	22	2 - Femini
2 - Discord	2 - Discord	3 - Concor	3 - Concor	3 - Concor	2 - Discord	Paciente	Não se apl	74	1 - Masculi
3 - Concor	3 - Concor	3 - Concor	3 - Concor	3 - Concor	3 - Concor	Cuidador	Familiar	65	1 - Masculi
3 - Concor	3 - Concor	2 - Discord	4 - Concor	4 - Concor	3 - Concor	Paciente	Não se apl	37	2 - Femini
3 - Concor	3 - Concor	3 - Concor	3 - Concor	3 - Concor	2 - Discord	Cuidador	Familiar	91	1 - Masculi
4 - Concor	4 - Concor	2 - Discord	4 - Concor	4 - Concor	2 - Discord	Paciente	Não se apl	78	2 - Femini
3 - Concor	4 - Concor	3 - Concor	3 - Concor	4 - Concor	3 - Concor	Paciente	Não se apl	58	1 - Masculi
2 - Discord	3 - Concor	3 - Concor	4 - Concor	4 - Concor	2 - Discord	Paciente	Não se apl	63	2 - Femini
4 - Concor	4 - Concor	2 - Discord	4 - Concor	4 - Concor	3 - Concor	Cuidador	Familiar	42	2 - Femini
2 - Discord	2 - Discord	2 - Discord	3 - Concor	3 - Concor	2 - Discord	Paciente	Não se apl	51	2 - Femini
3 - Concor	3 - Concor	2 - Discord	3 - Concor	3 - Concor	3 - Concor	Paciente	Não se apl	40	1 - Masculi
4 - Concor	4 - Concor	0 - Não sei	4 - Concor	3 - Concor	0 - Não sei	Cuidador	Familiar	75	1 - Masculi
2 - Discord	2 - Discord	2 - Discord	3 - Concor	3 - Concor	3 - Concor	Paciente	Não se apl	49	1 - Masculi
4 - Concor	4 - Concor	4 - Concor	4 - Concor	4 - Concor	4 - Concor	Paciente	Não se apl	99 - Entrev	99 - Entrev
3 - Concor	3 - Concor	4 - Concor	3 - Concor	3 - Concor	3 - Concor	Cuidador	Profissiona	57	1 - Masculi
3 - Concor	3 - Concor	3 - Concor	3 - Concor	3 - Concor	3 - Concor	Paciente	Não se apl	70	1 - Masculi
4 - Concor	2 - Discord	2 - Discord	3 - Concor	3 - Concor	2 - Discord	Cuidador	Familiar	53	1 - Masculi
3 - Concor	3 - Concor	3 - Concor	3 - Concor	3 - Concor	3 - Concor	Cuidador	Familiar	75	1 - Masculi
3 - Concor	3 - Concor	2 - Discord	3 - Concor	3 - Concor	3 - Concor	Paciente	Profissiona	61	1 - Masculi
4 - Concor	4 - Concor	4 - Concor	4 - Concor	4 - Concor	3 - Concor	Cuidador	Familiar	81	2 - Femini
2 - Discord	3 - Concor	1 - Discord	4 - Concor	3 - Concor	1 - Discord	Paciente	Não se apl	39	2 - Femini
3 - Concor	3 - Concor	3 - Concor	3 - Concor	3 - Concor	3 - Concor	Paciente	Não se apl	99 - Entrev	99 - Entrev
4 - Concor	4 - Concor	4 - Concor	4 - Concor	3 - Concor	1 - Discord	Paciente	Não se apl	37	2 - Femini
4 - Concor	4 - Concor	0 - Não sei	4 - Concor	4 - Concor	4 - Concor	Paciente	Não se apl	40	1 - Masculi
3 - Concor	3 - Concor	2 - Discord	3 - Concor	2 - Discord	3 - Concor	Cuidador	Familiar	24	1 - Masculi
3 - Concor	3 - Concor	2 - Discord	3 - Concor	3 - Concor	3 - Concor	Paciente	Não se apl	46	1 - Masculi
3 - Concor	3 - Concor	3 - Concor	3 - Concor	3 - Concor	3 - Concor	Cuidador	Familiar	58	1 - Masculi
3 - Concor	2 - Discord	2 - Discord	3 - Concor	3 - Concor	3 - Concor	Paciente	Não se apl	62	2 - Femini
3 - Concor	3 - Concor	3 - Concor	3 - Concor	3 - Concor	3 - Concor	Cuidador	Familiar	99 - Entrev	99 - Entrev
3 - Concor	3 - Concor	3 - Concor	3 - Concor	3 - Concor	2 - Discord	Paciente	Não se apl	34	1 - Masculi

3 - Concor	3 - Concor	2 - Discord	2 - Discord	2 - Discord	2 - Discord	Paciente	Profissiona	34	1 - Masculi
3 - Concor	3 - Concor	2 - Discord	3 - Concor	3 - Concor	3 - Concor	Cuidador	Familiar	97	2 - Feminir
4 - Concor	3 - Concor	4 - Concor	3 - Concor	3 - Concor	2 - Discord	Paciente	Profissiona	91	2 - Feminir
2 - Discord	2 - Discord	4 - Concor	4 - Concor	4 - Concor	2 - Discord	Paciente	Profissiona	54	2 - Feminir
4 - Concor	4 - Concor	4 - Concor	4 - Concor	4 - Concor	4 - Concor	Paciente	Não se apl	62	2 - Feminir
3 - Concor	3 - Concor	3 - Concor	3 - Concor	3 - Concor	3 - Concor	Paciente	Não se apl	44	2 - Feminir
4 - Concor	4 - Concor	3 - Concor	4 - Concor	3 - Concor	3 - Concor	Paciente	Não se apl	75	2 - Feminir
2 - Discord	3 - Concor	2 - Discord	3 - Concor	3 - Concor	3 - Concor	Cuidador	Familiar	57	2 - Feminir
4 - Concor	3 - Concor	2 - Discord	4 - Concor	4 - Concor	3 - Concor	Cuidador	Profissiona	44	1 - Masculi
3 - Concor	3 - Concor	4 - Concor	3 - Concor	3 - Concor	3 - Concor	Cuidador	Familiar	66	1 - Masculi
3 - Concor	3 - Concor	3 - Concor	3 - Concor	3 - Concor	3 - Concor	Paciente	Não se apl	65	1 - Masculi
4 - Concor	4 - Concor	4 - Concor	4 - Concor	4 - Concor	4 - Concor	Paciente	Não se apl	54	1 - Masculi
3 - Concor	3 - Concor	1 - Discord	3 - Concor	3 - Concor	2 - Discord	Paciente	Não se apl	65	1 - Masculi
3 - Concor	3 - Concor	3 - Concor	3 - Concor	3 - Concor	2 - Discord	Cuidador	Familiar	92	2 - Feminir
4 - Concor	4 - Concor	4 - Concor	4 - Concor	4 - Concor	4 - Concor	Paciente	Não se apl	64	2 - Feminir
2 - Discord	3 - Concor	3 - Concor	3 - Concor	3 - Concor	2 - Discord	Cuidador	Familiar	68	1 - Masculi
1 - Discord	2 - Discord	4 - Concor	4 - Concor	4 - Concor	4 - Concor	Paciente	Profissiona	46	1 - Masculi
3 - Concor	3 - Concor	4 - Concor	3 - Concor	3 - Concor	3 - Concor	Paciente	Não se apl	30	2 - Feminir
3 - Concor	3 - Concor	3 - Concor	3 - Concor	3 - Concor	3 - Concor	Paciente	Não se apl	61	2 - Feminir
2 - Discord	2 - Discord	2 - Discord	3 - Concor	3 - Concor	2 - Discord	Cuidador	Familiar	73	1 - Masculi
4 - Concor	2 - Discord	3 - Concor	3 - Concor	4 - Concor	2 - Discord	Paciente	Não se apl	99 - Entrev	99 - Entrev
4 - Concor	3 - Concor	2 - Discord	4 - Concor	4 - Concor	2 - Discord	Paciente	Não se apl	45	99 - Entrev
4 - Concor	4 - Concor	4 - Concor	4 - Concor	4 - Concor	4 - Concor	Paciente	Não se apl	45	99 - Entrev
3 - Concor	3 - Concor	2 - Discord	2 - Discord	2 - Discord	2 - Discord	Cuidador	Familiar	55	2 - Feminir
3 - Concor	3 - Concor	3 - Concor	3 - Concor	3 - Concor	2 - Discord	Paciente	Não se apl	36	1 - Masculi

Qual é o se	Você sabe	Qual é a su	Quantas pe	Quantos di	Quantos di	Você fez u	Você possu	Você possu	Hipertensã
4 - Ensino	1 - Branca	2 - R$2.09	3	15	2	0 - Não	3 - Ex-fum	1 - Sim	1 - Sim
3 - Ensino	1 - Branca	6 - Prefiro	2	20	0	0 - Não	3 - Ex-fum	1 - Sim	1 - Sim
4 - Ensino	1 - Branca	2 - R$2.09	2	7	0	0 - Não	3 - Ex-fum	1 - Sim	1 - Sim
2 - Ensino	1 - Branca	2 - R$2.09	3	5	0	0 - Não	3 - Ex-fum	0 - Não	0 - Não
5 - Ensino	1 - Branca	1 - Até R$2	4	9	0	0 - Não	1 - Não fur	0 - Não	0 - Não
5 - Ensino	3 - Parda	2 - R$2.09	2	25	15	1 - Sim	1 - Não fur	0 - Não	0 - Não
5 - Ensino	1 - Branca	2 - R$2.09	4	7	0	0 - Não	1 - Não fur	0 - Não	0 - Não
3 - Ensino	2 - Preta	2 - R$2.09	2	4	0	0 - Não	3 - Ex-fum	0 - Não	1 - Sim
3 - Ensino	3 - Parda	1 - Até R$2	4	21	15	1 - Sim	3 - Ex-fum	0 - Não	1 - Sim
6 - Ensino	1 - Branca	3 - R$5.22	4	8	0	0 - Não	1 - Não fur	1 - Sim	0 - Não
3 - Ensino	3 - Parda	2 - R$2.09	3	4	0	0 - Não	3 - Ex-fum	0 - Não	0 - Não
7 - Ensino	1 - Branca	3 - R$5.22	5	37	33	1 - Sim	1 - Não fur	0 - Não	1 - Sim
3 - Ensino	1 - Branca	1 - Até R$2	4	38	23	1 - Sim	3 - Ex-fum	1 - Sim	0 - Não
3 - Ensino	3 - Parda	99 - Entrev	99 - Entrev	99 - Entrev	99 - Entrev	99 - Entrev	99 - Entrev	99 - Entrev	99 - Entrev
5 - Ensino	3 - Parda	2 - R$2.09	2	21	9	1 - Sim	3 - Ex-fum	0 - Não	1 - Sim
99 - Entrev	99 - Entrev	99 - Entrev	99 - Entrev	99 - Entrev	99 - Entrev	99 - Entrev	99 - Entrev	99 - Entrev	99 - Entrev
7 - Ensino	1 - Branca	2 - R$2.09	1	7	0	0 - Não	3 - Ex-fum	0 - Não	0 - Não
7 - Ensino	1 - Branca	6 - Prefiro	2	25	24	1 - Sim	1 - Não fur	0 - Não	0 - Não
2 - Ensino	2 - Preta	2 - R$2.09	3	3	0	0 - Não	3 - Ex-fum	0 - Não	0 - Não
7 - Ensino	1 - Branca	6 - Prefiro	1	3	0	0 - Não	3 - Ex-fum	0 - Não	1 - Sim
3 - Ensino	1 - Branca	2 - R$2.09	3	21	14	1 - Sim	1 - Não fur	0 - Não	0 - Não
4 - Ensino	1 - Branca	2 - R$2.09	2	5	0	0 - Não	3 - Ex-fum	0 - Não	0 - Não
5 - Ensino	1 - Branca	1 - Até R$2	2	23	0	0 - Não	1 - Não fur	0 - Não	0 - Não
5 - Ensino	1 - Branca	2 - R$2.09	3	20	4	1 - Sim	1 - Não fur	0 - Não	0 - Não
2 - Ensino	3 - Parda	1 - Até R$2	4	12	0	0 - Não	3 - Ex-fum	1 - Sim	0 - Não
3 - Ensino	3 - Parda	2 - R$2.09	2	12	3	0 - Não	3 - Ex-fum	0 - Não	0 - Não
7 - Ensino	1 - Branca	3 - R$5.22	2	24	18	1 - Sim	1 - Não fur	1 - Sim	0 - Não
2 - Ensino	3 - Parda	2 - R$2.09	3	15	0	0 - Não	3 - Ex-fum	0 - Não	1 - Sim
2 - Ensino	1 - Branca	6 - Prefiro	3	105	30	1 - Sim	3 - Ex-fum	0 - Não	1 - Sim
5 - Ensino	1 - Branca	2 - R$2.09	5	7	0	0 - Não	2 - Fumant	1 - Sim	1 - Sim
5 - Ensino	1 - Branca	3 - R$5.22	2	27	10	1 - Sim	1 - Não fur	0 - Não	1 - Sim
5 - Ensino	1 - Branca	3 - R$5.22	3	25	17	1 - Sim	3 - Ex-fum	0 - Não	0 - Não
2 - Ensino	1 - Branca	1 - Até R$2	5	4	0	0 - Não	1 - Não fur	0 - Não	1 - Sim
2 - Ensino	1 - Branca	2 - R$2.09	3	13	0	0 - Não	3 - Ex-fum	0 - Não	1 - Sim
5 - Ensino	1 - Branca	2 - R$2.09	2	11	7	0 - Não	3 - Ex-fum	0 - Não	1 - Sim
5 - Ensino	1 - Branca	6 - Prefiro	4	16	0	0 - Não	1 - Não fur	0 - Não	1 - Sim
99 - Entrev	99 - Entrev	99 - Entrev	99 - Entrev	99 - Entrev	99 - Entrev	99 - Entrev	99 - Entrev	99 - Entrev	99 - Entrev
3 - Ensino	1 - Branca	2 - R$2.09	5	7	0	0 - Não	1 - Não fur	0 - Não	1 - Sim
5 - Ensino	3 - Parda	6 - Prefiro	3	10	0	0 - Não	3 - Ex-fum	0 - Não	0 - Não
3 - Ensino	1 - Branca	2 - R$2.09	3	11	0	0 - Não	3 - Ex-fum	0 - Não	0 - Não
1 - Sem ins	1 - Branca	1 - Até R$2	1	14	3	0 - Não	1 - Não fur	0 - Não	1 - Sim
6 - Ensino	1 - Branca	2 - R$2.09	4	30	26	1 - Sim	3 - Ex-fum	1 - Sim	0 - Não
4 - Ensino	1 - Branca	2 - R$2.09	5	14	0	0 - Não	1 - Não fur	1 - Sim	0 - Não
7 - Ensino	1 - Branca	2 - R$2.09	4	7	0	0 - Não	1 - Não fur	0 - Não	1 - Sim
99 - Entrev	99 - Entrev	99 - Entrev	99 - Entrev	99 - Entrev	99 - Entrev	99 - Entrev	99 - Entrev	99 - Entrev	99 - Entrev
6 - Ensino	1 - Branca	5 - Sem re	3	55	20	1 - Sim	1 - Não fur	0 - Não	0 - Não
5 - Ensino	1 - Branca	2 - R$2.09	3	3	0	0 - Não	1 - Não fur	0 - Não	1 - Sim
4 - Ensino	1 - Branca	1 - Até R$2	3	9	9	0 - Não	1 - Não fur	0 - Não	0 - Não
5 - Ensino	2 - Preta	2 - R$2.09	3	23	8	1 - Sim	1 - Não fur	0 - Não	0 - Não
1 - Sem ins	3 - Parda	1 - Até R$2	2	60	20	1 - Sim	3 - Ex-fum	0 - Não	1 - Sim
5 - Ensino	1 - Branca	2 - R$2.09	1	4	0	0 - Não	2 - Fumant	1 - Sim	1 - Sim
99 - Entrev	99 - Entrev	99 - Entrev	99 - Entrev	99 - Entrev	99 - Entrev	99 - Entrev	99 - Entrev	99 - Entrev	99 - Entrev
5 - Ensino	2 - Preta	2 - R$2.09	2	5	0	0 - Não	1 - Não fur	1 - Sim	0 - Não

2 - Ensino	1 - Branca	4 - Mais de	3	7	3	0 - Não	1 - Não fur	0 - Não	0 - Não
2 - Ensino	2 - Preta	2 - R$2.09	3	11	0	0 - Não	1 - Não fur	1 - Sim	1 - Sim
3 - Ensino	1 - Branca	2 - R$2.09	1	6	0	0 - Não	3 - Ex-fum	0 - Não	1 - Sim
2 - Ensino	3 - Parda	2 - R$2.09	4	13	8	1 - Sim	3 - Ex-fum	0 - Não	1 - Sim
2 - Ensino	1 - Branca	2 - R$2.09	2	25	7	1 - Sim	3 - Ex-fum	0 - Não	1 - Sim
2 - Ensino	1 - Branca	2 - R$2.09	4	45	20	1 - Sim	1 - Não fur	0 - Não	1 - Sim
2 - Ensino	1 - Branca	2 - R$2.09	2	5	0	0 - Não	1 - Não fur	0 - Não	1 - Sim
5 - Ensino	1 - Branca	3 - R$5.22	4	4	3	0 - Não	1 - Não fur	0 - Não	0 - Não
5 - Ensino	1 - Branca	3 - R$5.22	4	8	0	0 - Não	1 - Não fur	0 - Não	0 - Não
6 - Ensino	2 - Preta	2 - R$2.09	4	10	0	0 - Não	1 - Não fur	0 - Não	1 - Sim
2 - Ensino	1 - Branca	1 - Até R$2	2	13	0	0 - Não	3 - Ex-fum	0 - Não	0 - Não
3 - Ensino	1 - Branca	2 - R$2.09	4	4	0	0 - Não	3 - Ex-fum	0 - Não	1 - Sim
7 - Ensino	1 - Branca	2 - R$2.09	3	4	0	0 - Não	2 - Fumant	0 - Não	1 - Sim
5 - Ensino	1 - Branca	1 - Até R$2	1	5	0	0 - Não	1 - Não fur	1 - Sim	0 - Não
6 - Ensino	1 - Branca	2 - R$2.09	1	15	10	1 - Sim	3 - Ex-fum	1 - Sim	0 - Não
5 - Ensino	1 - Branca	2 - R$2.09	2	25	0	0 - Não	3 - Ex-fum	0 - Não	0 - Não
5 - Ensino	1 - Branca	2 - R$2.09	6	16	0	0 - Não	1 - Não fur	0 - Não	1 - Sim
5 - Ensino	1 - Branca	1 - Até R$2	2	32	22	1 - Sim	1 - Não fur	1 - Sim	0 - Não
6 - Ensino	1 - Branca	1 - Até R$2	4	15	3	0 - Não	3 - Ex-fum	1 - Sim	1 - Sim
6 - Ensino	1 - Branca	1 - Até R$2	1	20	7	0 - Não	3 - Ex-fum	1 - Sim	0 - Não
99 - Entrev	99 - Entrev	99 - Entrev	99 - Entrev	99 - Entrev	99 - Entrev	99 - Entrev	99 - Entrev	99 - Entrev	99 - Entrev
99 - Entrev	99 - Entrev	99 - Entrev	99 - Entrev	99 - Entrev	99 - Entrev	99 - Entrev	99 - Entrev	99 - Entrev	99 - Entrev
99 - Entrev	99 - Entrev	99 - Entrev	99 - Entrev	7	0	99 - Entrev	99 - Entrev	99 - Entrev	99 - Entrev
5 - Ensino	1 - Branca	2 - R$2.09	3	15	9	1 - Sim	1 - Não fur	0 - Não	1 - Sim
6 - Ensino	3 - Parda	1 - Até R$2	2	32	24	1 - Sim	3 - Ex-fum	0 - Não	0 - Não

Doenças C	Diabetes M	Doenças R	Obesidade	Câncer?	Durante o	Fadiga?	Falta de ar	Tosse?	Perda de o
0 - Não	0 - Não	0 - Não	1 - Sim	0 - Não	0 - Não	1 - Sim	1 - Sim	0 - Não	0 - Não
1 - Sim	0 - Não	0 - Não	1 - Sim	0 - Não	1 - Sim	1 - Sim	1 - Sim	1 - Sim	1 - Sim
0 - Não	0 - Não	0 - Não	1 - Sim	0 - Não	1 - Sim	1 - Sim	0 - Não	0 - Não	0 - Não
1 - Sim	0 - Não	1 - Sim	0 - Não	0 - Não	1 - Sim	1 - Sim	1 - Sim	1 - Sim	0 - Não
0 - Não	0 - Não	0 - Não	0 - Não	0 - Não	1 - Sim	0 - Não	0 - Não	0 - Não	0 - Não
0 - Não	0 - Não	0 - Não	0 - Não	0 - Não	1 - Sim	1 - Sim	1 - Sim	1 - Sim	0 - Não
0 - Não	0 - Não	0 - Não	0 - Não	0 - Não	1 - Sim	1 - Sim	1 - Sim	1 - Sim	0 - Não
1 - Sim	0 - Não	0 - Não	1 - Sim	0 - Não	0 - Não	1 - Sim	1 - Sim	1 - Sim	1 - Sim
0 - Não	1 - Sim	0 - Não	0 - Não	0 - Não	1 - Sim	1 - Sim	1 - Sim	1 - Sim	0 - Não
0 - Não	0 - Não	0 - Não	1 - Sim	0 - Não	1 - Sim	1 - Sim	1 - Sim	1 - Sim	1 - Sim
0 - Não	0 - Não	0 - Não	1 - Sim	0 - Não	1 - Sim	1 - Sim	1 - Sim	1 - Sim	0 - Não
0 - Não	0 - Não	0 - Não	0 - Não	0 - Não	1 - Sim	1 - Sim	1 - Sim	1 - Sim	1 - Sim
0 - Não	0 - Não	0 - Não	0 - Não	0 - Não	1 - Sim	1 - Sim	1 - Sim	1 - Sim	0 - Não
99 - Entrev	99 - Entrev	99 - Entrev	99 - Entrev	99 - Entrev	99 - Entrev	99 - Entrev	99 - Entrev	99 - Entrev	99 - Entrev
0 - Não	1 - Sim	0 - Não	0 - Não	0 - Não	1 - Sim	1 - Sim	1 - Sim	1 - Sim	0 - Não
99 - Entrev	99 - Entrev	99 - Entrev	99 - Entrev	99 - Entrev	99 - Entrev	99 - Entrev	99 - Entrev	99 - Entrev	99 - Entrev
0 - Não	0 - Não	0 - Não	0 - Não	0 - Não	1 - Sim	1 - Sim	1 - Sim	1 - Sim	0 - Não
0 - Não	0 - Não	0 - Não	0 - Não	0 - Não	1 - Sim	1 - Sim	1 - Sim	1 - Sim	0 - Não
0 - Não	1 - Sim	0 - Não	0 - Não	0 - Não	1 - Sim	1 - Sim	0 - Não	1 - Sim	1 - Sim
0 - Não	1 - Sim	1 - Sim	0 - Não	1 - Sim	1 - Sim	1 - Sim	0 - Não	0 - Não	0 - Não
0 - Não	0 - Não	0 - Não	1 - Sim	0 - Não	1 - Sim	1 - Sim	1 - Sim	1 - Sim	0 - Não
0 - Não	1 - Sim	0 - Não	0 - Não	0 - Não	1 - Sim	1 - Sim	1 - Sim	1 - Sim	1 - Sim
0 - Não	0 - Não	1 - Sim	0 - Não	1 - Sim	1 - Sim	1 - Sim	1 - Sim	1 - Sim	1 - Sim
0 - Não	0 - Não	0 - Não	1 - Sim	0 - Não	1 - Sim	1 - Sim	1 - Sim	1 - Sim	1 - Sim
1 - Sim	0 - Não	1 - Sim	0 - Não	0 - Não	0 - Não	1 - Sim	1 - Sim	0 - Não	0 - Não
0 - Não	0 - Não	0 - Não	0 - Não	0 - Não	1 - Sim	1 - Sim	1 - Sim	1 - Sim	0 - Não
0 - Não	0 - Não	0 - Não	0 - Não	0 - Não	1 - Sim	1 - Sim	1 - Sim	1 - Sim	0 - Não
0 - Não	0 - Não	0 - Não	0 - Não	0 - Não	1 - Sim	1 - Sim	1 - Sim	1 - Sim	0 - Não
0 - Não	0 - Não	0 - Não	0 - Não	0 - Não	0 - Não	1 - Sim	1 - Sim	1 - Sim	0 - Não
1 - Sim	1 - Sim	0 - Não	0 - Não	0 - Não	1 - Sim	1 - Sim	1 - Sim	1 - Sim	0 - Não
0 - Não	0 - Não	0 - Não	0 - Não	0 - Não	1 - Sim	1 - Sim	1 - Sim	0 - Não	0 - Não
0 - Não	0 - Não	1 - Sim	0 - Não	0 - Não	1 - Sim	1 - Sim	1 - Sim	0 - Não	1 - Sim
1 - Sim	1 - Sim	0 - Não	1 - Sim	1 - Sim	0 - Não	1 - Sim	1 - Sim	0 - Não	1 - Sim
1 - Sim	1 - Sim	0 - Não	1 - Sim	0 - Não	1 - Sim	1 - Sim	1 - Sim	1 - Sim	1 - Sim
0 - Não	1 - Sim	0 - Não	0 - Não	0 - Não	1 - Sim	1 - Sim	1 - Sim	1 - Sim	1 - Sim
0 - Não	0 - Não	1 - Sim	0 - Não	0 - Não	1 - Sim	0 - Não	0 - Não	0 - Não	0 - Não
99 - Entrev	99 - Entrev	99 - Entrev	99 - Entrev	99 - Entrev	99 - Entrev	99 - Entrev	99 - Entrev	99 - Entrev	99 - Entrev
0 - Não	1 - Sim	0 - Não	0 - Não	1 - Sim	1 - Sim	1 - Sim	1 - Sim	1 - Sim	1 - Sim
0 - Não	0 - Não	0 - Não	0 - Não	0 - Não	1 - Sim	1 - Sim	1 - Sim	0 - Não	1 - Sim
0 - Não	0 - Não	0 - Não	0 - Não	0 - Não	1 - Sim	1 - Sim	1 - Sim	0 - Não	0 - Não
0 - Não	1 - Sim	0 - Não	0 - Não	1 - Sim	0 - Não	0 - Não	0 - Não	0 - Não	0 - Não
0 - Não	0 - Não	0 - Não	0 - Não	0 - Não	1 - Sim	1 - Sim	1 - Sim	0 - Não	0 - Não
1 - Sim	0 - Não	0 - Não	0 - Não	0 - Não	1 - Sim	1 - Sim	1 - Sim	1 - Sim	1 - Sim
0 - Não	0 - Não	0 - Não	1 - Sim	0 - Não	1 - Sim	1 - Sim	1 - Sim	1 - Sim	1 - Sim
99 - Entrev	99 - Entrev	99 - Entrev	99 - Entrev	99 - Entrev	99 - Entrev	99 - Entrev	99 - Entrev	99 - Entrev	99 - Entrev
0 - Não	0 - Não	0 - Não	0 - Não	0 - Não	1 - Sim	1 - Sim	1 - Sim	1 - Sim	0 - Não
0 - Não	0 - Não	0 - Não	0 - Não	0 - Não	1 - Sim	1 - Sim	1 - Sim	1 - Sim	1 - Sim
0 - Não	0 - Não	0 - Não	1 - Sim	0 - Não	1 - Sim	1 - Sim	1 - Sim	1 - Sim	0 - Não
0 - Não	0 - Não	0 - Não	0 - Não	0 - Não	1 - Sim	1 - Sim	1 - Sim	1 - Sim	1 - Sim
0 - Não	1 - Sim	0 - Não	0 - Não	0 - Não	1 - Sim	1 - Sim	1 - Sim	1 - Sim	0 - Não
1 - Sim	0 - Não	0 - Não	1 - Sim	0 - Não	0 - Não	1 - Sim	1 - Sim	1 - Sim	0 - Não
99 - Entrev	99 - Entrev	99 - Entrev	99 - Entrev	99 - Entrev	99 - Entrev	99 - Entrev	99 - Entrev	99 - Entrev	99 - Entrev
0 - Não	0 - Não	0 - Não	0 - Não	0 - Não	1 - Sim	1 - Sim	1 - Sim	1 - Sim	0 - Não

0 - Não	0 - Não	0 - Não	1 - Sim	0 - Não	1 - Sim	1 - Sim	0 - Não	1 - Sim	1 - Sim
1 - Sim	0 - Não	0 - Não	0 - Não	0 - Não	0 - Não	1 - Sim	1 - Sim	0 - Não	0 - Não
0 - Não	0 - Não	0 - Não	0 - Não	0 - Não	1 - Sim	1 - Sim	0 - Não	1 - Sim	1 - Sim
0 - Não	1 - Sim	0 - Não	1 - Sim	0 - Não	0 - Não	1 - Sim	1 - Sim	0 - Não	1 - Sim
0 - Não	1 - Sim	0 - Não	0 - Não	0 - Não	1 - Sim	1 - Sim	1 - Sim	1 - Sim	1 - Sim
0 - Não	0 - Não	0 - Não	0 - Não	0 - Não	1 - Sim	1 - Sim	1 - Sim	1 - Sim	0 - Não
1 - Sim	1 - Sim	0 - Não	0 - Não	0 - Não	0 - Não	1 - Sim	1 - Sim	1 - Sim	1 - Sim
0 - Não	0 - Não	0 - Não	1 - Sim	1 - Sim	1 - Sim	1 - Sim	0 - Não	1 - Sim	1 - Sim
0 - Não	0 - Não	1 - Sim	1 - Sim	0 - Não	1 - Sim	1 - Sim	1 - Sim	1 - Sim	1 - Sim
0 - Não	0 - Não	0 - Não	0 - Não	0 - Não	1 - Sim	1 - Sim	1 - Sim	1 - Sim	0 - Não
0 - Não	0 - Não	0 - Não	1 - Sim	0 - Não	1 - Sim	1 - Sim	0 - Não	1 - Sim	0 - Não
0 - Não	1 - Sim	0 - Não	1 - Sim	1 - Sim	0 - Não	1 - Sim	1 - Sim	1 - Sim	1 - Sim
0 - Não	0 - Não	0 - Não	0 - Não	0 - Não	1 - Sim	1 - Sim	1 - Sim	1 - Sim	0 - Não
1 - Sim	0 - Não	0 - Não	0 - Não	0 - Não	1 - Sim	1 - Sim	1 - Sim	1 - Sim	1 - Sim
0 - Não	0 - Não	0 - Não	0 - Não	0 - Não	1 - Sim	1 - Sim	1 - Sim	0 - Não	0 - Não
0 - Não	1 - Sim	1 - Sim	0 - Não	0 - Não	1 - Sim	1 - Sim	1 - Sim	0 - Não	1 - Sim
0 - Não	0 - Não	0 - Não	1 - Sim	0 - Não	1 - Sim	1 - Sim	1 - Sim	1 - Sim	1 - Sim
0 - Não	0 - Não	0 - Não	1 - Sim	0 - Não	1 - Sim	1 - Sim	1 - Sim	1 - Sim	0 - Não
1 - Sim	0 - Não	0 - Não	0 - Não	0 - Não	1 - Sim	1 - Sim	1 - Sim	1 - Sim	1 - Sim
1 - Sim	1 - Sim	0 - Não	0 - Não	0 - Não	1 - Sim	1 - Sim	0 - Não	1 - Sim	0 - Não
99 - Entrev	99 - Entrev	99 - Entrev	99 - Entrev	99 - Entrev	99 - Entrev	99 - Entrev	99 - Entrev	99 - Entrev	99 - Entrev
99 - Entrev	99 - Entrev	99 - Entrev	99 - Entrev	99 - Entrev	99 - Entrev	99 - Entrev	99 - Entrev	99 - Entrev	99 - Entrev
99 - Entrev	99 - Entrev	99 - Entrev	99 - Entrev	99 - Entrev	99 - Entrev	99 - Entrev	99 - Entrev	99 - Entrev	99 - Entrev
1 - Sim	1 - Sim	0 - Não	0 - Não	0 - Não	1 - Sim	1 - Sim	1 - Sim	1 - Sim	1 - Sim
0 - Não	0 - Não	0 - Não	1 - Sim	0 - Não	1 - Sim	1 - Sim	1 - Sim	1 - Sim	1 - Sim

Dor de cab	Dor no cor|	Náuseas e	Diarreia?
0 - Não	0 - Não	0 - Não	0 - Não
1 - Sim	0 - Não	0 - Não	0 - Não
1 - Sim	1 - Sim	1 - Sim	1 - Sim
1 - Sim	1 - Sim	0 - Não	0 - Não
1 - Sim	1 - Sim	1 - Sim	1 - Sim
0 - Não	0 - Não	0 - Não	0 - Não
1 - Sim	1 - Sim	1 - Sim	1 - Sim
1 - Sim	1 - Sim	0 - Não	0 - Não
1 - Sim	1 - Sim	1 - Sim	1 - Sim
1 - Sim	1 - Sim	0 - Não	1 - Sim
1 - Sim	1 - Sim	1 - Sim	1 - Sim
1 - Sim	1 - Sim	0 - Não	0 - Não
0 - Não	0 - Não	0 - Não	0 - Não
99 - Entrev	99 - Entrev	99 - Entrev	99 - Entrevista suspensa
1 - Sim	1 - Sim	1 - Sim	1 - Sim
99 - Entrev	99 - Entrev	99 - Entrev	99 - Entrevista suspensa
1 - Sim	1 - Sim	0 - Não	0 - Não
0 - Não	0 - Não	0 - Não	0 - Não
0 - Não	1 - Sim	1 - Sim	1 - Sim
0 - Não	1 - Sim	0 - Não	1 - Sim
0 - Não	1 - Sim	0 - Não	0 - Não
0 - Não	1 - Sim	0 - Não	0 - Não
1 - Sim	1 - Sim	0 - Não	0 - Não
1 - Sim	1 - Sim	0 - Não	1 - Sim
0 - Não	1 - Sim	0 - Não	0 - Não
1 - Sim	1 - Sim	1 - Sim	1 - Sim
0 - Não	1 - Sim	0 - Não	0 - Não
0 - Não	1 - Sim	0 - Não	0 - Não
0 - Não	1 - Sim	1 - Sim	1 - Sim
1 - Sim	1 - Sim	0 - Não	1 - Sim
0 - Não	0 - Não	0 - Não	0 - Não
1 - Sim	1 - Sim	0 - Não	1 - Sim
1 - Sim	1 - Sim	1 - Sim	0 - Não
0 - Não	1 - Sim	0 - Não	1 - Sim
1 - Sim	1 - Sim	1 - Sim	1 - Sim
0 - Não	0 - Não	1 - Sim	0 - Não
99 - Entrev	99 - Entrev	99 - Entrev	99 - Entrevista suspensa
1 - Sim	1 - Sim	1 - Sim	1 - Sim
0 - Não	1 - Sim	1 - Sim	0 - Não
0 - Não	1 - Sim	1 - Sim	1 - Sim
0 - Não	0 - Não	0 - Não	0 - Não
1 - Sim	1 - Sim	0 - Não	0 - Não
0 - Não	0 - Não	0 - Não	1 - Sim
1 - Sim	1 - Sim	1 - Sim	1 - Sim
99 - Entrev	99 - Entrev	99 - Entrev	99 - Entrevista suspensa
1 - Sim	1 - Sim	1 - Sim	1 - Sim
1 - Sim	1 - Sim	1 - Sim	1 - Sim
1 - Sim	1 - Sim	0 - Não	1 - Sim
1 - Sim	1 - Sim	0 - Não	1 - Sim
0 - Não	1 - Sim	1 - Sim	1 - Sim
0 - Não	1 - Sim	0 - Não	1 - Sim
99 - Entrev	99 - Entrev	99 - Entrev	99 - Entrevista suspensa
1 - Sim	1 - Sim	1 - Sim	1 - Sim

1 - Sim	1 - Sim	1 - Sim	0 - Não
1 - Sim	1 - Sim	0 - Não	0 - Não
1 - Sim	1 - Sim	1 - Sim	0 - Não
0 - Não	1 - Sim	0 - Não	1 - Sim
0 - Não	1 - Sim	1 - Sim	0 - Não
1 - Sim	1 - Sim	1 - Sim	0 - Não
0 - Não	0 - Não	0 - Não	0 - Não
1 - Sim	1 - Sim	1 - Sim	1 - Sim
1 - Sim	1 - Sim	0 - Não	1 - Sim
1 - Sim	1 - Sim	1 - Sim	1 - Sim
1 - Sim	1 - Sim	0 - Não	1 - Sim
1 - Sim	1 - Sim	0 - Não	1 - Sim
0 - Não	0 - Não	0 - Não	1 - Sim
0 - Não	1 - Sim	0 - Não	0 - Não
1 - Sim	1 - Sim	0 - Não	0 - Não
0 - Não	1 - Sim	1 - Sim	1 - Sim
1 - Sim	1 - Sim	0 - Não	0 - Não
1 - Sim	1 - Sim	0 - Não	0 - Não
1 - Sim	1 - Sim	1 - Sim	0 - Não
1 - Sim	1 - Sim	0 - Não	0 - Não
99 - Entrev	99 - Entrev	99 - Entrev	99 - Entrevista suspensa
99 - Entrev	99 - Entrev	99 - Entrev	99 - Entrevista suspensa
99 - Entrev	99 - Entrev	99 - Entrev	99 - Entrevista suspensa
1 - Sim	1 - Sim	0 - Não	0 - Não
1 - Sim	1 - Sim	1 - Sim	0 - Não
