# Supplementary material for: Quality of Care Transition for COVID-19 Patients in a University Hospital in Southern Brazil
Source: Rev Bras Enferm. 2024 Jun 28;77(Suppl 1):e20230402. doi: 10.1590/0034-7167-2023-0402 (PMC11213544; doi:10.1590/0034-7167-2023-0402)
Supplement: Translation

# Translation: Qualidade da transição do cuidado de pacientes com covid-19 em um hospital universitário ao sul do Brasil

**DOI:** 10.1590/0034-7167-2023-0402pt

**Authors:** Ana Cecilia Boeng, Caroline Cechinel-Peiter, Maria Fernanda Baeta Neves Alonso da Costa, Laísa Fisher Wachholz, José Luís Guedes dos Santos, Gabriela Marcellino de Melo Lanzoni

**Affiliations:** I Universidade Federal de Santa Catarina. Florianópolis, Santa Catarina, Brasil

**Keywords:** Cuidado Transicional, Covid-19, Alta do Paciente, Continuidade da Assistência ao Paciente, Hospitais Universitários

## Abstract

**Objetivo:**

Avaliar a qualidade da transição do cuidado do hospital para o domicílio de pacientes com covid-19.

**Método:**

Estudo transversal, realizado em um Hospital Universitário no Sul do Brasil, com 78 pacientes que tiveram alta hospitalar após internação por covid-19. A coleta de dados foi realizada por telefone e utilizou a versão brasileira do *Care Transitions Measure* (CTM-15). Os dados foram analisados por meio de estatística descritiva e analítica.

**Resultados:**

A média da qualidade da transição do cuidado foi de 70,8, em uma escala que varia de zero a 100, indicando uma qualidade moderada da transição do cuidado. O maior escore foi atribuído ao fator 1, “Preparação para autogerenciamento”, e o menor ao fator 4, “Plano de cuidado”.

**Conclusões:**

É importante aprimorar a comunicação e o suporte oferecidos aos pacientes durante o processo de transição, especialmente no que diz respeito à compreensão dos medicamentos prescritos e ao desenvolvimento de planos de cuidados claros.

## INTRODUÇÃO

A pandemia abalou a capacidade assistencial dos sistemas de saúde, trazendo consequências drásticas na área social, econômica e na saúde da população^(1)^. Anteriormente à pandemia, o momento da transição do paciente entre os serviços de saúde já correspondia a um período propício à ruptura da continuidade do cuidado^(2)^. O processo de alta hospitalar, por sua vez, é complexo, e a transição do paciente para o domicílio representa o momento em que o paciente está mais propenso a eventos adversos^(3)^.

A crise sanitária ocasionada pela pandemia exigiu maior cautela no acompanhamento dos pacientes após a alta hospitalar, devido à complexidade e particularidade dos cuidados após a alta que o paciente hospitalizado pela covid-19 necessita. A maioria dos pacientes não retorna às suas condições prévias de saúde no momento da alta, geralmente necessitando de acompanhamento especializado após a internação^(4)^. Estudos constataram declínio funcional e aumento da fragilidade em pelo menos um terço dos pacientes em até 3 meses após a internação por covid-19.

Tais condições estão relacionadas a uma maior mortalidade e diminuição da qualidade de vida dos pacientes, além de representarem uma maior responsabilidade para os familiares, cuidadores e para o sistema de saúde como um todo. Estas evidências enfatizam o impacto gerado pela doença nos pacientes e suas famílias mesmo após a internação, reforçando a importância do acompanhamento, plano de cuidados personalizado e reabilitação^(5-6)^.

É comum pacientes vítimas da covid-19 receberem alta para a casa com lacunas na transição do cuidado, tendo como consequência o aumento da incidência de complicações, readmissões e a falta de seguimento no cuidado. Fatores como determinantes sociais, de saúde e condições de moradia influenciam na capacidade dos pacientes e famílias de seguirem acompanhamento extra-hospitalar após a internação^(7)^.

As ações relacionadas à transição do cuidado visam garantir a transferência segura do paciente entre os serviços de saúde, por meio de modelos assistenciais que assegurem a continuidade do cuidado^(2)^. A transição do cuidado constitui uma importante estratégia para a efetivação de um sistema integrado de saúde, sendo uma forma de superar a fragmentação da atenção e garantir a continuidade dos cuidados na rede de atenção à saúde^(8)^.

O enfermeiro é o principal profissional responsável por proporcionar transições bem-sucedidas, garantindo que os pacientes voltem ao domicílio preparados e com o apoio adequado^(9)^. Devido à sua experiência e competência no trabalho em equipe, no manejo de pacientes complexos e suas famílias, o enfermeiro é o principal articulador entre profissionais e serviços dos diferentes níveis de atenção, geralmente assumindo o papel do planejamento para a alta, promovendo ações e interações entre profissionais e serviços, pacientes e cuidadores, e assim visando assegurar a continuidade dos cuidados após a alta hospitalar^(3,10)^.

Entre os principais benefícios de transições do cuidado efetivas estão a redução das reinternações e visitas ao setor de emergência, principalmente em pacientes portadores de doenças crônicas e com idade avançada. A diminuição da mortalidade, dos custos hospitalares, de eventos adversos e aumento da qualidade de vida e satisfação dos pacientes também são benefícios já evidenciados nos estudos^(3)^.

Diversas questões podem influenciar a qualidade e a experiência da transição do cuidado do paciente, entre elas as suas expectativas, seu nível de conhecimento, autonomia, bem-estar emocional e físico, além do ambiente de cuidado e nível de planejamento da transição^(2)^. Compreender a percepção dos pacientes e cuidadores acerca do processo de transição do cuidado é essencial para a elaboração de estratégias que contribuam para o desenvolvimento e implementação de transições mais qualificadas^(8)^ e se torna ainda mais relevante devido à escassez de estudos internacionais descrevendo a qualidade da transição do cuidado do paciente após a internação por covid-19.

Nesse sentido, com o intuito de auxiliar profissionais e gestores de saúde a identificar aspectos a serem melhorados na transição do cuidado, questiona-se: como é avaliada a qualidade, na perspectiva de pacientes e cuidadores, da transição do cuidado de pacientes com covid-19 a partir da alta de um Hospital Universitário ao sul do Brasil?

## OBJETIVO

Avaliar a qualidade da transição do cuidado do hospital para o domicílio de pacientes com covid-19.

## MÉTODOS

### Aspectos éticos

A pesquisa seguiu as determinações da Resolução nº 466, de 12 de dezembro de 2012 do Conselho Nacional de Saúde, que dispõe sobre os cuidados da pesquisa com seres humanos. O estudo foi aprovado pelo Comitê de Ética em Pesquisa da Universidade Federal de Santa Catarina. Como a coleta de dados foi desenvolvida via chamada telefônica, o consentimento ocorreu de forma verbal, obtido no momento da chamada, sendo gravado e devidamente arquivado sob custódia dos pesquisadores.

### Desenho, período e local do estudo

Este estudo é parte da pesquisa multicêntrica intitulada “Avaliação do cuidado de enfermagem a pacientes com covid-19 em hospitais universitários brasileiros”, financiada pelo Conselho Nacional de Desenvolvimento Científico e Tecnológico (CNPq), por meio da chamada pública MCTIC/CNPq/FNDCT/MS/SCTIE/Decit Nº 07/2020 - Pesquisas para enfrentamento da covid-19, suas consequências e outras síndromes respiratórias agudas graves. O estudo seguiu o guia para reportar pesquisas observacionais STROBE.

A coleta foi realizada no período de abril a dezembro de 2021, período caracterizado por aumento acentuado no número de casos e óbitos por covid-19 no contexto nacional. O campo de estudo foi um Hospital Universitário ao Sul do Brasil administrado pela Empresa Brasileira de Serviços Hospitalares (EBSERH), escolhido por ser referência para o atendimento em saúde para a região. Trata-se de um hospital geral de grande porte com 245 leitos. Foram inseridos no estudo apenas as unidades de internação nas quais os profissionais participaram do atendimento de pacientes adultos com suspeita ou diagnóstico confirmado de covid-19 e realizaram a alta para casa. Destaca-se que a instituição não possuía serviço especializado para apoio ao processo de transição do cuidado. Enfermeiros e médicos eram os principais responsáveis por orientar pacientes e familiares, bem como fornecer documentos, como sumário de alta e prescrição de medicamentos.

### População ou amostra

A população do estudo foi composta por pacientes e cuidadores que tiveram alta para casa após internação por covid-19. Foram considerados os critérios de inclusão: idade superior a 18 anos, fluência no idioma português do brasil, internação de no mínimo 72 horas para atendimento específico de covid-19, e alta hospitalar em um período superior a sete dias, contados no momento da ligação telefônica.

A amostra mínima foi calculada no programa *Winpepi*, versão 11.65, a partir do número de leitos do hospital, utilizando uma margem de erro de 4 pontos, desvio padrão de 17,1 e nível de confiança de 95%. Desta forma, foi estimada uma amostra mínima de 63 pacientes.

### Protocolo do estudo

A coleta de dados foi realizada por ligações telefônicas, considerando o período de sete dias após a alta do paciente. Foram utilizados dois instrumentos: 1) instrumento de caracterização; e 2) versão brasileira do *Care Transitions Measure* (CTM-15). O instrumento de caracterização incluiu as variáveis: idade; gênero; grau de instrução; raça; renda familiar; tempo total de internação; tempo de internação em UTI; uso de ventilação mecânica invasiva; histórico de tabagismo; sintomas apresentados, e comorbidades.

O CTM-15 é um instrumento desenvolvido nos Estados Unidos em 2002 que tem como objetivo avaliar a qualidade da transição do cuidado entre os diferentes serviços de atenção à saúde segundo a perspectiva do paciente. O instrumento é formado por 15 afirmações sobre o processo de transição do cuidado que são divididas em 4 fatores: Preparação para autogerenciamento; Entendimento sobre medicações; Preferências asseguradas e Plano de cuidado. Essas afirmações são medidas por uma escala Likert com cinco opções de respostas que variam de “concordo muito” a “discordo muito” ^(8)^.

### Análise dos resultados

Os dados foram tabulados em uma planilha do programa Excel e analisados pelo software *Statistical Package for the Social Science* (SPSS) versão 25. As variáveis categóricas foram apresentadas por meio de frequência absoluta e relativa, e as variáveis contínuas por meio de medidas de tendência central e de dispersão.

Para obtenção dos escores e análise da qualidade da transição do cuidado, foram calculados a soma dos valores referentes às respostas: Discordo muito (1 ponto); Discordo (2 pontos); Concordo (3 pontos); e Concordo muito (4 pontos). A opção “Não sei/não lembro/não se aplica” corresponde a 0 pontos e é analisada separadamente, não entrando na contagem.

O escore obtido pela soma dos valores referentes às respostas foi dividido pelo número de questões respondidas de 1 a 4 pontos. Este resultado foi transformado, então, em uma escala linear de zero a 100, conforme orientações dos autores do instrumento^(11)^. A média da escala entre todos os participantes foi calculada, indicando o nível de qualidade da transição do cuidado naquele contexto.

A média da pontuação do CTM-15 também foi testada frente às variáveis independentes, utilizando-se os testes não paramétricos de Mann-Whitney, Kruskal-Wallis e correlação de Spearman. O nível de significância considerado foi de 95% (p<0,05).

## RESULTADOS

Participaram da pesquisa 78 pacientes que tiveram alta hospitalar após internação por covid-19. Em 54 (69,2%) entrevistas, foram os pacientes que responderam o instrumento. Em relação às características sociodemográficas, a maioria dos participantes era homem (57,7%), com média de idade de 53,7 anos (dp 17,6), ensino fundamental completo (43,7%), raça branca (74,6%), com renda familiar entre dois e cinco salários mínimos (62,5%). O tempo de internação dos pacientes variou de três a 105 dias, com uma média de 16,7 dias (dp=16), e o tempo de internação em UTI foi de zero a 33 dias (média 6 dias; dp=8,9). A maioria dos pacientes não necessitou de ventilação mecânica invasiva. Com relação ao histórico de tabagismo, menos da metade dos participantes relatou nunca ter fumado. Quanto às comorbidades, a condição mais prevalente foi a Hipertensão Arterial Sistêmica, seguida de Obesidade e Diabetes Mellitus. A maioria dos pacientes apresentou fadiga, febre, dispneia, artralgia e mialgia, tosse e cefaleia pelo menos uma vez durante a internação (Tabela 1).

**Tabela 1:** Caracterização sociodemográfica e clínica dos participantes incluídos no estudo. Florianópolis, Santa Catarina, Brasil, 2022

Variáveis	n	%
Gênero n=71		
Masculino	41	57,7
Feminino	30	42,3
Grau de instrução n=71		
Ensino Fundamental	31	43,7
Ensino Médio	23	32,4
Ensino Superior	15	21,1
Sem instrução	2	2,8
Raça n=71		
Branca	53	74,6
Parda	12	16,9
Preta	6	8,5
Outra	-	-
Renda familiar n=64		
2 a 5 salários mínimos	40	62,5
Até 2 salários mínimos	15	23,4
Mais de 5 salários mínimos	8	12,5
Sem rendimentos	1	1,6
Uso de ventilação mecânica n=70		
Não	47	67,1
Sim	23	32,9
Histórico de tabagismo n=70		
Ex-fumante	34	48,6
Não fumante	33	47,1
Fumante	3	4,3
Comorbidades n=70		
Hipertensão Arterial Sistêmica	34	48,6
Obesidade	22	31,4
Diabetes Mellitus	19	27,1
Doença respiratória crônica	18	25,7
Doenças Cardiovasculares	15	21,4
Doenças renais	8	11,4
Câncer	7	10,0
Sinais e sintomas n=70		
Fadiga	67	95,7
Febre	59	84,3
Dispneia	59	84,3
Mialgia e artralgia	59	84,3
Tosse	53	75,7
Cefaleia	43	61,4
Diarreia	35	50,0
Anosmia e ageusia	32	45,7
Náuseas e vômitos	29	41,4

A média total do CTM-15 foi 70,8 (dp=17,0). O maior escore (79,1) foi atribuído ao item 4, “Teve informações que precisava para autocuidado”. Os itens 6, relacionado ao entendimento de sinais de alerta e sintomas, e os itens 13 e 14, ambos relacionados ao entendimento do paciente sobre a terapia medicamentosa, apresentaram-se na sequência. No entanto, o item 15, “entendimento sobre os efeitos colaterais”, apresentou o menor escore (Tabela 2).

**Tabela 2:** Qualidade da transição do cuidado segundo os itens do instrumento *Care Transitions Measure* (CTM-15). Florianópolis, Santa Catarina, Brasil, 2022

Item		Fator	Média	Desvio Padrão
4	Teve informações que precisava para autocuidado	1	79,1	24,4
6	Entende sinais de alerta e sintomas	1	77,9	19,2
14	Entende como tomar os medicamentos	2	77,5	18,3
13	Entende o motivo de tomar os medicamentos	2	77,5	19,1
9	Compreende o que é de sua responsabilidade	1	76,5	20,9
1	Concordou com a equipe de saúde sobre objetivos para sua saúde e como eles seriam alcançados	3	75,6	20,8
8	Compreende o que melhora ou piora sua condição de saúde	1	75,6	21,3
5	Entende claramente como cuidar da saúde	1	75,1	23,3
10	Sente-se seguro de que sabe o que fazer	1	71,5	22,9
2	Preferências consideradas para decidir as necessidades de saúde	3	71,1	20,8
11	Sente-se seguro de que consegue fazer o que é necessário	1	71,0	22,5
3	Preferências consideradas para decidir onde as necessidades de saúde são atendidas	3	68,5	23,3
7	Recebeu um plano escrito de cuidados	4	67,6	26,4
12	Recebeu uma lista escrita das consultas ou exames	4	62,3	25,5
15	Entende os efeitos colaterais dos medicamentos	2	60,2	24,2

Dentre os fatores do CTM-15, podemos observar que o maior escore foi atribuído ao fator 1, “Preparação para autogerenciamento”, e o menor ao fator 4, “Plano de cuidado” (Tabela 3).

**Tabela 3:** Qualidade da transição do cuidado segundo os Fatores do instrumento *Care Transitions Measure* (CTM-15). Florianópolis, Santa Catarina, Brasil, 2022

Fator		Média	Desvio Padrão
1	Preparação para o autogerenciamento	74,1	19,4
2	Entendimento sobre medicações	70,8	17,3
3	Preferências asseguradas	69,6	21,3
4	Plano de cuidado	60,2	25,1

Foram correlacionadas a qualidade da transição do cuidado e as características sociodemográficas dos participantes do estudo. Nesta análise, destacou-se a relação segundo raça, em que participantes autodeclarados pardos atribuíram valores menores à qualidade da transição do cuidado, especialmente ao que se refere ao fator 3 do CTM-15, referente às preferências asseguradas (Tabela 4).

**Tabela 4:** Qualidade da transição do cuidado segundo os Fatores do instrumento *Care Transitions Measure* (CTM-15) e as características sociodemográficas dos participantes. Florianópolis, Santa Catarina, Brasil, 2022

	Fator 1	Fator 2	Fator 3	Fator 4	Total
	**Média (dp)**	**Média (dp)**	**Média (dp)**	**Média (dp)**	**Média (dp)**
Gênero					
Feminino	75,1 (22,1)	70,9 (18,2)	71,9 (22,4)	60,0 (26,5)	71,5 (18,7)
Masculino	72,1 (18,1)	69,5 (17,1)	66,5 (20,8)	58,3 (24,5)	68,9 (15,8)
Valor de *p*	0,446	0,622	0,266	0,822	0,533
Grau de instrução					
Sem instrução	66,7 -	66,7 -	66,7 -	66,7 -	66,7 -
Ensino Fundamental	77,3 (16,8)	69,6(18,7)	70,6(22,1)	60,2 (24,2)	72,2 (16,2)
Ensino Médio	70,4 (23,6)	73,2 (18,4)	65,7 (19,5)	62,9 (25,7)	69,2 (18,8)
Ensino Superior	70,8 (20,4)	66,7 (14,6)	70,0 (25,2)	50,0 (27,5)	67,2 (17,7)
Valor de *p*	0,419	0,537	0,894	0,468	0,750
Raça					
Branca	73,9 (21,4)	71,2 (18,2)	70,6 (22,6)	59,0 (28,5)	70,9 (18,4)
Preta	77,0 (17,9)	70,4 (15,2)	75,9 (19,1)	58,3 (13,9)	72,8 (13,4)
Parda	69,1 (12,6)	64,7 (14,8)	57,4 (13,3)	59,7 (11,1)	64,5 (10,7)
Valor de *p*	0,684	0,505	0,047	0,960	0,443
Renda familiar					
Até 2 SM^*^	65,4 (20,7)	68,9 (16,9)	63,0 (16,6)	58,9 (18,8)	64,9 (16,0)
2 a 5 SM^*^	77,1 (16,7)	70,7 (18,5)	70,3 (22,0)	63,3 (28,4)	72,8 (16,6)
Mais de 5 SM^*^	78,6 (21,6)	75,0 (19,5)	79,2 (21,0)	47,9 (24,3)	73,9 (18,9)
Sem rendimentos	100 -	55,6 -	100 -	66,7 -	86,7 -
Valor de *p*	0,157	0,489	0,128	0,514	0,424

* *SM= Salários Mínimos.*

Em relação às características clínicas dos participantes, nota-se que um maior escore foi atribuído pelos participantes que apresentaram os sintomas de dispneia, tosse, anosmia e ageusia durante a infecção pela covid-19 (Tabela 5).

**Tabela 5:** Qualidade da transição do cuidado segundo os Fatores do instrumento *Care Transitions Measure* (CTM-15) e as características clínicas dos participantes. Florianópolis, Santa Catarina, Brasil, 2022

	Fator 1	Fator 2	Fator 3	Fator 4	Total
	Média (dp)	Média (dp)	Média (dp)	Média (dp)	Média (dp)
Uso de ventilação mecânica					
Não	71,6 (20,1)	68,9 (16,9)	65,6 (22,6)	55,8 (26,6)	68,0 (17,2)
Sim	76,8 (17,3)	72,5 (18,6)	76,8 (16,0)	65,9 (21,6)	74,5 (14,7)
Valor de *p*	0,427	0,314	0,047	0,079	0,116
Histórico de tabagismo					
Não fumante	71,0 (21,2)	69,4 (18,2)	72,7 (18,0)	59,6 (28,0)	69,5 (17,2)
Fumante	73,0 (16,7)	66,7 (11,1)	66,7 (11,1)	44,4 (34,7)	67,3 (14,9)
Ex-fumante	75,6 (19,2)	71,1 (17,4)	66,2 (24,4)	60,1 (22,0)	71,0 (17,6)
Valor de *p*	0,569	0,970	0,539	0,578	0,758
Doença respiratória crônica					
Não	73,7 (20,8)	70,0 (17,9)	68,7 (22,1)	57,5 (26,1)	70,0 (17,4)
Sim	72,2 (17,8)	70,4 (16,6)	71,0 (18,7)	63,9 (23,0)	77,6 (16,5)
Valor de *p*	0,588	0,972	0,917	0,462	0,840
Hipertensão Arterial Sistêmica					
Não	72,4 (22,2)	70,1 (18,9)	67,6 (23,4)	60,2 (24,3)	69,5 (19,2)
Sim	74,4 (17,5)	70,1 (16,0)	71,1 (18,8)	58,1 (26,7)	70,8 (14,8)
Valor de *p*	0,762	0,520	0,526	0,768	0,851
Doenças Cardiovasculares					
Não	75,0 (19,9)	72,0 (18,1)	70,2 (21,2)	61,7 (25,4)	71,8 (17,1)
Sim	67,3 (19,5)	63,0 (13,1)	65,9 (21,6)	50,0 (23,6)	64,1 (16,2)
Valor de *p*	0,087	0,112	0,444	0,088	0,088
Diabetes Mellitus					
Não	72,6 (19,4)	68,4 (16,7)	69,7 (18,7)	56,9 (25,6)	68,2 (16,1)
Sim	75,4 (21,6)	74,6 (19,0)	68,1 (27,3)	65,7 (23,9)	72,7 (19,8)
Valor de *p*	0,622	0,118	0,734	0,269	0,358
Doenças Renais					
Não	74,4 (18,8)	69,8 (17,5)	71,9 (18,0)	61,5 (25,2)	71,3 (16,2)
Sim	65,5 (27,2)	72,2 (17,8)	49,3 (33,3)	41,7 (19,9)	61,1 (22,3)
Valor de *p*	0,264	0,856	0,063	0,038	0,142
Obesidade					
Não	73,9 (21,0)	71,4 (17,5)	69,1 (22,9)	61,0 (23,6)	70,9 (17,6)
Sim	72,1 (17,8)	67,2 (17,3)	69,7 (17,6)	55,3 (28,8)	68,5 (16,1)
Valor de *p*	0,853	0,340	0,933	0,259	0,539
Câncer					
Não	74,0 (19,1)	69,6 (17,5)	70,2 (19,8)	59,4 (24,6)	70,6 (16,3)
Sim	67,4 (27,1)	74,6 (17,8)	61,1 (31,9)	57,1 (33,1)	66,3 (24,3)
Valor de *p*	0,515	0,418	0,409	0,676	0,543
Febre					
Não	73,6 (21,3)	68,7 (14,8)	65,7 (23,0)	57,6 (20,2)	69,0 (17,9)
Sim	73,3 (19,8)	70,3 (18,0)	70,0 (21,0)	59,5 (26,3)	70,4 (17,1)
Valor de *p*	0,961	0,987	0,523	0,687	0,710
Fadiga					
Não	41,3 (24,0)	66,7 -	59,3 (12,8)	44,4 (19,3)	50,4 (15,6)
Sim	74,8 (18,7)	70,2 (17,8)	69,7 (21,5)	59,9 (25,5)	71,0 (16,7)
Valor de *p*	0,019	0,978	0,326	0,271	0,067
Dispneia					
Não	58,9 (23,5)	64,7 (15,6)	63,1 (24,4)	43,9 (17,1)	59,1 (17,6)
Sim	76,0 (18,2)	71,1 (17,7)	70,4 (20,6)	62,1 (25,7)	72,2 (16,3)
Valor de *p*	0,025	0,473	0,249	0,018	0,032
Tosse					
Não	63,0 (22,3)	67,3 (13,3)	54,6 (24,9)	54,9 (19,3)	61,2 (18,0)
Sim	76,6 (18,1)	71,0 (18,6)	74,0 (17,7)	60,6 (27,0)	73,0 (16,0)
Valor de *p*	0,040	0,646	0,003	0,336	0,024
Anosmia e ageusia					
Não	68,7 (18,7)	66,7 (14,6)	64,8 (19,1)	57,0 (22,1)	66,2 (15,3)
Sim	78,9 (20,1)	74,1 (19,7)	74,7 (22,6)	61,8 (28,9)	74,8 (18,2)
Valor de *p*	0,027	0,073	0,024	0,545	0,030
Cefaleia					
Não	71,4 (19,5)	70,0 (17,1)	65,2 (24,0)	59,9 (20,8)	68,5 (17,2)
Sim	74,5 (20,3)	70,2 (17,8)	71,8 (19,2)	58,7 (28,1)	71,2 (17,2)
Valor de *p*	0,563	0,896	0,340	0,880	0,566
Mialgia e artralgia					
Não	64,5 (20,0)	66,7 (17,9)	67,7 (16,8)	54,6 (23,7)	64,2 (16,6)
Sim	75,0 (19,6)	70,7 (17,4)	69,6 (22,0)	60,1 (25,7)	71,2 (17,1)
Valor de *p*	0,145	0,438	0,578	0,440	0,180
Náuseas e vômitos					
Não	74,1 (18,2)	71,0 (17,9)	70,9 (20,5)	58,9 (25,9)	71,0 (16,5)
Sim	72,3 (22,4)	68,8 (17,0)	67,1 (22,3)	59,5 (25,0)	68,9 (18,1)
Valor de *p*	0,805	0,644	0,384	0,703	0,694
Diarreia					
Não	69,5 (18,5)	66,7 (18,9)	68,6 (20,3)	56,7 (24,0)	67,1 (16,4)
Sim	77,1 (20,9)	73,5 (15,4)	70,0 (22,4)	61,8 (26,8)	73,1 (17,5)
Valor de *p*	0,081	0,065	0,550	0,305	0,115

Foi observado um menor escore no fator 1 “Preparação para autogerenciamento” nos pacientes que apresentaram fadiga, em relação àqueles que negaram esse sintoma (41,3 para 74,8, respectivamente) e aos demais grupos. O menor escore do fator 2 “Entendimento sobre medicações” foi atribuído pelos participantes com doenças cardiovasculares (63,0). Os fatores 3 “Preferências asseguradas” e 4 “Plano de cuidado” obtiveram escores significantemente inferiores entre os pacientes que possuíam doenças renais (49,3 e 41,7, respectivamente) em comparação aos que não possuíam (71,9 e 61,5, respectivamente), sendo evidenciada uma diferença estatística significativa na atribuição dos escores do fator 4 nos pacientes que possuíam ou não essa comorbidade (p=0,038). É notável também uma diferença estatística significativa nos escores do fator 1 e 3 (p=0,040 e p=0,003) em relação aos pacientes que apresentaram ou não tosse como sintomatologia e nos escores do fator 1 entre os pacientes que apresentaram ou não anosmia e ageusia (p=0,027).

## DISCUSSÃO

Os resultados desta pesquisa permitiram a avaliação da transição do cuidado em pacientes internados por covid-19. A média de escore do CTM-15 neste estudo foi de 70,8 (dp 17,0), o que demonstra uma qualidade moderada da transição do cuidado. Na interpretação do instrumento, uma média de escores elevados indica uma percepção positiva dos pacientes acerca da transição do cuidado. Na literatura, não é descrito um ponto de corte para os escores do CTM-15; no entanto, estudos realizados utilizando o CTM-15 em pacientes adultos no Brasil e Estados Unidos evidenciam escores que variam de 69,5 a 78,5^(8,12-14)^. Tendo em vista o contexto pandêmico, com superlotação de hospitais, sobrecarga de profissionais e escassez de recursos, considera-se uma qualidade moderada da transição do cuidado como um resultado positivo.

Devido à natureza atual da doença, foram localizados poucos estudos descrevendo a experiência de transição do cuidado de pacientes que foram internados por covid-19. Um estudo conduzido na Espanha acerca das experiências de pacientes hospitalizados pela covid-19 demonstrou que, apesar da gratidão e positividade acerca do cuidado recebido, muitos pacientes sentiram que nem sempre receberam o cuidado que deveriam devido à falta de recursos materiais e humanos causados pela pandemia^(15)^. Já um estudo conduzido no Canadá demonstrou que a maioria dos pacientes sentiu que sua alta foi apressada e que não estavam bem preparados e informados para ir para casa. Alguns relataram não ter recebido as informações e orientações necessárias no momento da alta, como contatos de apoio e aconselhamento, e um plano de cuidado, e que, por isso, muitas vezes tiveram que retornar à emergência do hospital. Pacientes também associaram essas lacunas no processo de transição e no cuidado como um todo ao estado de pandemia e aos protocolos de restrição vigentes^(16)^.

O maior escore do estudo foi para o fator 1 “Preparação para autogerenciamento”, em especial ao item 4, “Teve informações que precisava para autocuidado”, o que demonstra um bom compartilhamento e entendimento de informações durante a hospitalização, assim como segurança dos pacientes para o autogerenciamento das suas necessidades de saúde no domicílio. Tal achado é positivo, tendo em vista que o preparo do paciente para o autogerenciamento da condição de saúde e autocuidado no domicílio está entre as intervenções mais efetivas para a construção de transições do cuidado bem qualificadas^(17)^.

O segundo maior escore do estudo foi atribuído ao fator 2, Entendimento sobre medicações. No entanto, o item 15, “Entende os efeitos colaterais dos medicamentos”, obteve o escore mais baixo, o que demonstra uma lacuna nas orientações fornecidas no momento da alta. Os resultados sugerem que informações sobre as medicações são fornecidas, porém os pacientes não são orientados acerca de efeitos colaterais, limitando-se a informações como dose, hora e via de administração^(12)^.

Orientações acerca do uso de medicações que incluam informações sobre os efeitos colaterais são fundamentais para evitar eventos adversos após a alta hospitalar^(3)^. As prescrições medicamentosas realizadas para a alta hospitalar apresentam um grande potencial problemático, especialmente devido à discrepância entre a terapia medicamentosa antes e após a hospitalização, visto que apenas 10% dos pacientes recebem alta com a mesma terapia medicamentosa da admissão^(18)^. Ações de reconciliação medicamentosa podem proporcionar resultados positivos nesse sentido. Estudos demonstraram uma queda no percentual de reinternações e diminuição de eventos adversos em pacientes que receberam esse tipo de intervenção, que também pode ser realizada por enfermeiros^(3,19)^.

O fator 4, Plano de cuidado, apresentou o menor escore do estudo. A pontuação dos itens 7 “Recebeu um plano escrito de cuidados” e 12 “Recebeu uma lista escrita das consultas ou exames” indicam que muitos pacientes tiveram alta sem receberem um plano de cuidados estruturado e encaminhamentos para seguimento pós-alta. Este achado é similar aos já descritos na literatura, e que demonstra uma fragilidade comum em diferentes serviços de saúde, e potencializada pelo contexto de pandemia. Ressalta-se que o planejamento e a construção de um plano de cuidados são essenciais para garantir o preparo do paciente para seu autogerenciamento no domicílio, enquanto os encaminhamentos para seguimento extra-hospitalar facilitam a absorção do paciente de forma responsável aos diferentes serviços de saúde, proporcionando a continuidade do cuidado^(12-13)^.

O seguimento ambulatorial permite o acompanhamento rigoroso e administração dos sintomas após a alta, permitindo que problemas e preocupações possam ser detectados e abordados precocemente, evitando reinternações desnecessárias. A comunicação entre os diferentes níveis de atenção auxilia na implementação de melhores práticas de transição e possibilita a continuidade do cuidado. No Brasil, no entanto, percebe-se uma fragmentação e falta de articulação dos hospitais com os demais níveis de atenção^(9,19)^.

O enfermeiro geralmente é responsável pela coordenação do cuidado, identificando necessidades e preferências, além de elaborar planos de cuidado individualizados. Esta prática acontece de forma mais delimitada em países onde existem enfermeiros direcionados especificamente às atividades de coordenação e planejamento da alta hospitalar, chamados de enfermeiros de ligação. Esses profissionais possuem um papel fundamental na continuidade do cuidado e são encarregados do planejamento da alta, educação em saúde, identificação de forças e fragilidades e a transferência de informações do paciente entre o hospital e os demais serviços de saúde^(20)^.

O estudo também constatou que pacientes que apresentaram sintomas como dispneia, tosse e anosmia/ageusia atribuíram melhores escores em comparação aos pacientes que não apresentaram esses sintomas, especialmente no que se refere ao fator 1, referente às ações de autogerenciamento da doença. Cabe destacar que estes sintomas estavam entre o rol dos principais sintomas associados aos agravos da covid-19 desde o surgimento da doença. Assim, estes achados podem estar relacionados ao fato de que estes sintomas representavam condições de alerta ao agravamento do quadro clínico, e assim, estes pacientes exigiam uma maior atenção do profissional no momento da transição do cuidado^(21)^.

Ainda quanto às características dos pacientes, foram constatados menores escores atrelados aos participantes de cor parda e com menor renda familiar. Pacientes de raça negra, em estudo conduzido nos Estados Unidos, relataram menos consultas de acompanhamento, menor probabilidade de receber um contato para sanar dúvidas e de receber equipamento para prosseguir com o cuidado em domicílio. Segundo os autores, tal discrepância pode estar relacionada a fatores como acesso e instrução em saúde, elementos também relacionados a limitações financeiras^(22)^. Contudo, os resultados deste estudo devem ser interpretados com cautela, uma vez que, apesar da diferença dos escores do CTM-15, a comparação entre as categorias não apresentou significância estatística, exigindo estudos aplicados a populações maiores para melhor compreensão do fenômeno.

O processo de transição do cuidado como um todo é complexo e envolve questões relacionadas à performance de profissionais, o uso de protocolos, entre outros. Dessa forma, avaliar a transição do cuidado em um contexto é um desafio que deve ser abordado de múltiplas perspectivas, instrumentos e indicadores^(12)^.

### Limitações do estudo

Este estudo apresenta algumas limitações que devem ser consideradas ao interpretar seus resultados. Primeiramente, o tamanho da amostra pode limitar a generalização dos achados para outras populações ou contextos de cuidado. Apesar de os participantes representarem uma amostra significativa, estudos com amostras maiores poderiam oferecer uma visão mais abrangente e robusta da qualidade da transição do cuidado em pacientes com covid-19.

Além disso, a natureza retrospectiva do estudo e a coleta de dados por meio de entrevistas telefônicas podem introduzir viéses de memória ou de resposta nos participantes, afetando a precisão e a confiabilidade dos dados coletados.

### Contribuições para a área da Enfermagem

Este estudo oferece contribuições significativas para a área da enfermagem ao destacar aspectos essenciais da transição do cuidado em pacientes hospitalizados por covid-19. Ao identificar os principais pontos fortes e fracos na qualidade da transição do cuidado, os enfermeiros podem direcionar intervenções específicas para melhorar a experiência e os resultados dos pacientes durante essa fase crítica.

Os achados destacam a necessidade de fornecer informações claras e abrangentes aos pacientes sobre autocuidado, medicação e sinais de alerta, visando capacitar os pacientes a assumir um papel ativo em sua recuperação após a alta hospitalar.

## CONCLUSÕES

Observou-se que a qualidade da transição do cuidado de pacientes internados pela covid-19 em um Hospital Universitário foi moderada. Aspectos relacionados às orientações recebidas durante a hospitalização, ao entendimento dos pacientes acerca destas e à incorporação das preferências dos pacientes no cuidado pós-alta apresentaram melhores escores. Já as principais fragilidades observadas foram referentes ao entendimento sobre os efeitos colaterais dos medicamentos, ao plano de cuidado e encaminhamentos pós-alta.

Por fim, mais investigações são necessárias para melhor avaliar as transições do cuidado, explorando diferentes perspectivas, determinantes sociais e de saúde e buscando estratégias com o objetivo de qualificar processos e ações nas instituições de saúde.

## Data Availability

https://doi.org/10.48331/scielodata.B4L4S8
